# BMC Cancer reviewer acknowledgement 2014

**DOI:** 10.1186/s12885-015-1049-8

**Published:** 2015-03-05

**Authors:** Dafne Solera

**Affiliations:** BioMed Central, Floor 6, 236 Gray’s Inn Road, London, WC1X 8HB United Kingdom

## Abstract

The editors of BMC Cancer would like to thank all our reviewers who have contributed to the journal in Volume 14 (2014).

**Philip Abbosh**

USA

**Daniel Abbott**

USA

**Sherif Abdel-Misih**

USA

**Omar Abdel-Wahab**

USA

**Takashige Abe**

Japan

**Heinz-Harald Abholz**

Germany

**Roger Abounader**

USA

**Lucas Abrahao-Machado**

Brazil

**Filippo Acconcia**

Italy

**Paolo Accornero**

Italy

**Christopher Adams**

USA

**Raffaele Addeo**

Italy

**Clement Adebamowo**

USA

**Prasad Adusumilli**

USA

**Joachim Aerts**

Netherlands

**Ilir Agalliu**

USA

**Kamran Aghayev**

USA

**Nishant Agrawal**

USA

**Yong Chan Ahn**

South Korea

**Hanjong Ahn**

South Korea

**Peter Ahn**

USA

**Myung-Ju Ahn**

South Korea

**Koichi Aiura**

Japan

**Sally Akarolo-Anthony**

USA

**Rosemary Akhurst**

USA

**Bülent Arman Aksoy**

USA

**Andre Albergaria**

Portugal

**Scott Albert**

USA

**Jennifer Aldrink**

USA

**Agustí Alentorn**

Spain

**Riccardo Alessandro**

Italy

**Michael Alexandrakis**

Greece

**Simak Ali**

UK

**Sadeer Al-Kindi**

USA

**Khawla Al-Kuraya**

Saudi Arabia

**Hatim Allawi**

USA

**Brian Allen**

USA

**Benjamin Allen**

USA

**Bryan Allen**

USA

**Michael Allen**

UK

**Matthew Allen**

USA

**Steven Allen**

UK

**Gabriela Almeida**

Portugal

**Redhwan Al-Naggar**

Malaysia

**Vicente Alonso**

Spain

**Sergio Alonso**

Spain

**Robert Alston**

UK

**Sean Altekruse**

USA

**Begona Alvarez-Gonzalez**

USA

**Antonio Alves**

Brazil

**Giulia Amadio**

Italy

**Larbi Amazit**

France

**Sreedhar Amere Subbarao**

India

**Arm Amin**

USA

**Albert Amini**

USA

**Ziv Amir**

UK

**Ping An**

USA

**Hee An**

South Korea

**Ming-Wen An**

USA

**Ruopeng An**

USA

**Ioannis Anagnostopoulos**

Germany

**Satoshi Anai**

Japan

**Venkateshwari Ananthapur**

India

**Dimitrios Anastasiou**

UK

**Berit Andersen**

Denmark

**Bogi Andersen**

USA

**Annie Anderson**

UK

**Benjamin Anderson**

USA

**Therese Andersson**

Sweden

**Bruno Andrade**

Brazil

**Victor Andrade**

Brazil

**Bengt Andrae**

Sweden

**Eran Andrechek**

USA

**Robert Andtbacka**

USA

**Mei-Kim Ang**

Singapore

**Jozef Anne**

Belgium

**Jean-Christophe Antoine**

France

**Alessandro Antonelli**

Italy

**Anji Anura**

India

**Daisuke Aoki**

Japan

**Lauren Aoude**

Australia

**Keishiro Aoyagi**

Japan

**Thomas Aparicio**

France

**Santiago Aparo**

USA

**Leonard Appleman**

USA

**Jack Arbiser**

USA

**Marc Arbyn**

Belgium

**Rosaria Arcone**

Italy

**Chantal Arditi**

Switzerland

**Diego Arenas**

Mexico

**Candy Arentz**

USA

**Paolo Aretini**

Italy

**David Argyle**

UK

**Saro Armenian**

USA

**Michael Arnold**

USA

**Manju Aron**

USA

**Amanda Arrington**

USA

**Hassan Arshad**

USA

**Robert Ashford**

UK

**Laura Ashley**

UK

**Jonathan Ashman**

USA

**Luigi Aurisicchio**

Italy

**Philippe Autier**

France

**Romane Auvergne**

USA

**Amir Avan**

Netherlands

**Kaamar Azijli**

Netherlands

**Hamdy Azim**

Egypt

**Amaya Azqueta**

Spain

**Maria Azrad**

USA

**Ligia Azzalis**

Brazil

**Tsukasa Baba**

Japan

**Hideo Baba**

Japan

**Matthias Bache**

Germany

**Ulrike Bacher**

Germany

**Floor Backes**

USA

**Celia Badenas**

Spain

**Sunil Badve**

USA

**Young Kyung Bae**

South Korea

**Jeong-Heum Baek**

South Korea

**Swapnil Bagade**

USA

**Cedo Bagi**

USA

**Hilary Bagshaw**

USA

**Bruce Baguley**

New Zealand

**Simin Bahrami**

USA

**Li Bai**

China

**Longchuan Bai**

USA

**Tibor Bakacs**

Hungary

**Paul Bakaki**

USA

**Megan Baker**

USA

**Debora Balabram**

Brazil

**Tessa Balach**

USA

**Claudia Baldus**

Germany

**Marija Balic**

Austria

**Balint László Balint**

Hungary

**Karla Ballman**

USA

**Zsolt Balogi**

USA

**Fatima Baltazar**

Portugal

**Hamid Band**

USA

**Vimla Band**

USA

**Aria Baniahmad**

Germany

**Zhu Baosong**

China

**Massimo Barberis**

Italy

**Dario Barbone**

USA

**Nuno L Barbosa-Morais**

Portugal

**Stephane Bardet**

France

**Debmalya Barh**

India

**David Barker**

USA

**Michele Barone**

Italy

**Richard Barr**

USA

**Carlos Barrios**

Brazil

**Richard Barron**

USA

**João Diogo Barros-Silva**

Portugal

**Twyla Bartel**

USA

**Alakananda Basu**

USA

**Devraj Basu**

USA

**Sandip Basu**

India

**Madhumita Basu**

USA

**Ana Battastini**

Brazil

**Adi Baumgartner**

UK

**Alessandra Bearz**

Italy

**Francisco Beca**

USA

**Laurent Bedenne**

France

**Alicia Beeghly-Fadiel**

USA

**Juergen Behrens**

Germany

**Tim Beissbarth**

Germany

**Tanios Bekaii-Saab**

USA

**Antonino Belfiore**

Italy

**Cristiana Bellan**

Italy

**Joaquim Bellmunt**

USA

**Qiwen Ben**

China

**Barbara Benassi**

Italy

**Offir Ben-Ishay**

Israel

**Lindsay Bennett**

UK

**Stephane Benoist**

France

**David Bentrem**

USA

**Carmen Berasain**

Spain

**Guy Berchem**

Luxembourg

**Giovanni Luca Beretta**

Italy

**Bernhard Berger**

Germany

**Vance Berger**

USA

**Thierry Berghmans**

Belgium

**Lothar Bergmann**

Germany

**Johannes Berkhof**

Netherlands

**Dominique Bernard-Gallon**

France

**Rene Bernards**

Netherlands

**Sonja Berndt**

USA

**Gilbert Bernier**

Canada

**Alfredo Berruti**

Italy

**Donald Berry**

USA

**Tiffany Berry**

USA

**Francois Bertucci**

France

**Marianne Berwick**

USA

**Tomoko Betsuyaku**

Japan

**Kishor Bhakat**

USA

**Castigliano Bhamidipati**

USA

**Uddalak Bharadwaj**

USA

**Anant Bhatt**

India

**Bhaskar Bhattacharya**

Singapore

**Malay Bhattacharyya**

India

**Ujjal Bhawal**

Japan

**Nirmala Bhoo Pathy**

Malaysia

**Feng Bi**

China

**Long Bi**

China

**Giampaolo Bianchini**

Italy

**Irene Biasoli**

Brazil

**Kai Bickenbach**

USA

**Hemant Kumar Bid**

USA

**Francois-Clement Bidard**

France

**Siamak Bidel**

Finland

**Ettore Bidoli**

Italy

**Silvia Bielsa**

Spain

**Bernhard Biersack**

Germany

**Giuseppe Bifulco**

Italy

**Irene Bijnsdorp**

Netherlands

**Andreas Bikfalvi**

France

**Kate Lynn Bill**

USA

**Thomas Binder**

Germany

**Rebecca Birch**

UK

**Sarah Birken**

USA

**Eszter Biro**

Hungary

**Anders Bjartell**

Sweden

**Kimberly Blackwell**

USA

**Eric Blair**

UK

**Carmen Blanco-Aparicio**

Spain

**Justin Blasberg**

USA

**Boris Blechacz**

USA

**Matthew Block**

USA

**Elisabeth Bloemena**

Netherlands

**Karen Blyth**

UK

**Zhai Bo**

China

**Gerd Bobe**

USA

**Carla Boccaccio**

Italy

**Udo Bode**

Germany

**Clara Bodelon**

USA

**Martin Bohac**

Slovakia

**Antonio Boing**

Brazil

**Richard Bold**

USA

**Jesper Bonde**

Denmark

**Edith Bonnelye**

France

**Stefanos Bonovas**

Greece

**Stephanie Boone**

USA

**Jan Bornschein**

Germany

**David Borowski**

UK

**Pedro Borralho**

Portugal

**Josep Borras**

Spain

**Stefania Bortoluzzi**

Italy

**Silvano Bosari**

Italy

**Fred Bosman**

Switzerland

**Paolo Bossi**

Italy

**Jan Bouchal**

Czech Republic

**Sotiria Boukouvala**

Greece

**Liam Bourke**

UK

**Anne-Marie Bouvier**

France

**Nathan Bowen**

USA

**John Boyages**

Australia

**Sabrina Boyrie**

USA

**Serdar Bozdag**

USA

**Alexandre Bozec**

France

**Katja Irene Braam**

Netherlands

**Edward Bradley**

Canada

**Lucia Braga**

Brazil

**Elena Ioana Braicu**

Germany

**Simone Braig**

Germany

**Kate Brain**

UK

**Mariana Brait**

USA

**Mirjana Brankovic-Magic**

Serbia

**Maria Braoudaki**

Greece

**Theodore Brasky**

USA

**Isabelle Bray**

UK

**Patrick Brest**

France

**Guy Brock**

USA

**Inka Brockhausen**

Canada

**Giuseppe Bronte**

Italy

**Reuben Broom**

New Zealand

**James Broome**

USA

**Christine Brostjan**

Austria

**Mick Brown**

UK

**Terry Brown**

UK

**Anthony Bruce**

Canada

**Adam Brufsky**

USA

**Matteo Brunelli**

Italy

**Lauro Bucchi**

Italy

**Jan Budczies**

Germany

**Laurien Maria Buffart**

Netherlands

**Olivier Buhard**

France

**Ralph Bundschuh**

Germany

**Emily Burger**

Norway

**Barbara Burtness**

USA

**Rita Busuttil**

Australia

**Sebastiano Buti**

Italy

**Lisa Butler**

Australia

**Alison Butt**

Australia

**Maria Cabanillas**

USA

**Hale Basak Caglar**

Turkey

**Mu-Yan Cai**

China

**Sanjun Cai**

China

**Dawei Cai**

USA

**Hong Cai**

China

**Silvia Calabuig-Fariñas**

Spain

**Natalia Calanzani**

UK

**George Calin**

USA

**Gloria Callard**

USA

**Federica Calore**

USA

**Enrica Calura**

Italy

**Felipe Calvo**

Spain

**E. Ramsay Camp**

USA

**Kerry Campbell**

USA

**Michael Campbell**

USA

**Kevin Camphausen**

USA

**Giuseppina Campisi**

Italy

**Nicole Campos**

USA

**Francisco Candido Dos Reis**

Brazil

**Qing Cao**

China

**Guangwen Cao**

China

**Wenqing Cao**

USA

**Gabriel Capella**

Spain

**Marzia Capelletti**

USA

**Vera Cappelletti**

Italy

**Emilia Caputo**

Italy

**Michele Caraglia**

Italy

**Eugen Carasevici**

Romania

**Romilda Cardin**

Italy

**Vinicio Carloni**

Italy

**Francisco Javier Carmona Sanz**

USA

**Shamus Carr**

USA

**Dirce Carraro**

Brazil

**Jason Carroll**

UK

**Rosangela Caruso**

Italy

**Luis Carvajal Carmona**

USA

**Beatriz Carvalho**

Netherlands

**Joana Carvalho**

Portugal

**Ivan Casaburi**

Italy

**Silvia Casadei**

USA

**Emilio Casanova**

Austria

**Patricia Casbas-Hernandez**

USA

**Bruce Case**

Canada

**Alejandra Castanon**

UK

**Daniel Castellano**

Spain

**Isabella Castellano**

Italy

**Gabriella Castoria**

Italy

**Stefania Catalano**

Italy

**Chelsea Catsburg**

USA

**Erika Cecchin**

Italy

**Francesco Cellini**

Italy

**Lorenzo Ceppi**

USA

**Giovanni Luca Ceresoli**

Italy

**Hyuk-Jin Cha**

South Korea

**Karl Chai**

USA

**Arthit Chairoungdua**

Thailand

**Marc Chamberlain**

USA

**Suzanne Chambers**

Australia

**Michael Chan**

Taiwan

**Jennifer Chan**

USA

**Jennifer Chan**

Canada

**Karen Chan**

Hong Kong

**Michael Chan**

USA

**Ashwini Chand**

Australia

**Alice Y.W. Chang**

Taiwan

**Chien-Chung Chang**

Taiwan

**Jong Hee Chang**

South Korea

**Kuo-Wei Chang**

Tanzania

**Yu-Chao Chang**

Taiwan

**Kai-Ping Chang**

Taiwan

**Hong Chang**

Canada

**Yu-Sun Chang**

Taiwan

**Yoon Soo Chang**

South Korea

**Wu Changjun**

China

**Jui-I Chao**

Taiwan

**Kathy Chapman**

Australia

**Bjoern Chapuy**

USA

**Al Charest**

USA

**Ayana Chase**

USA

**Arun Chauhan**

USA

**Alcides Chaux**

Paraguay

**Srikumar Chellappan**

USA

**Mu-Kuan Chen**

Taiwan

**Ching-Shih Chen**

USA

**Chunhua Chen**

China

**Chuang Chen**

China

**Chang-Han Chen**

Taiwan

**Ming Chen**

China

**Wan Qing Chen**

China

**Zhe-Sheng Chen**

USA

**Tse-Ching Chen**

Taiwan

**Honglei Chen**

China

**James Chen**

USA

**Jeon-Hor Chen**

USA

**Jianyong Chen**

USA

**Kuen-Feng Chen**

Taiwan

**Ying Chen**

China

**Wu-Fu Chen**

Taiwan

**Zhiyi Chen**

China

**Xinbin Chen**

USA

**Yongshun Chen**

China

**Yongwen Chen**

China

**Zong-You Chen**

China

**Chao-Wen Cheng**

Taiwan

**Ting-Yuan Cheng**

USA

**Jason Chia-Hsien Cheng**

Taiwan

**Steven Cheng**

China

**Dong-Joo Cheon**

USA

**Jaehun Cheong**

South Korea

**Sok Ching Cheong**

Malaysia

**Radostina Cherneva**

Bulgaria

**Chandramu Chetty**

USA

**Arthur Cheung**

Hong Kong

**Felix Cheung**

USA

**Karen Cheung**

Canada

**Siu Tim Cheung**

Hong Kong

**Chun-Ju Chiang**

Taiwan

**Hung-Che Chiang**

Taiwan

**Laura Chiecchio**

UK

**Chih-Yen Chien**

Taiwan

**Lung-Chang Chien**

USA

**Yoshitomo Chihara**

Japan

**Giovanna Chiorino**

Italy

**Yolanda I Chirino**

Mexico

**Tai-Jan Chiu**

Taiwan

**Sherry Yueh-Hsia Chiu**

Taiwan

**Miguel Chiurillo**

Venezuela

**Sang-Hee Cho**

South Korea

**Yung Hyun Choi**

South Korea

**Kyung-Chul Choi**

South Korea

**Kui Son Choi**

South Korea

**Lydia Choi**

USA

**Won-Il Choi**

South Korea

**Ravi Chokshi**

USA

**Geoffrey Chong**

Australia

**Su Pin Choo**

Singapore

**Helene Choquet**

USA

**Teh-Ying Chou**

Taiwan

**Kuan-Chih Chow**

Taiwan

**Louis Chow**

Hong Kong

**Balram Chowbay**

Singapore

**Edwin Choy**

USA

**Jesper Frank Christensen**

Denmark

**Saranya Chumsri**

USA

**Kuo-Piao Chung**

Taiwan

**Yeun-Jun Chung**

South Korea

**Younh-Hwa Chung**

South Korea

**Fabio Cianchi**

Italy

**Amelia Cimmino**

Italy

**Daniel Ciocca**

Argentina

**Paola Ciriaco**

Italy

**Frank Claessens**

Belgium

**Andrew Clamp**

UK

**Jennifer Clarke**

USA

**Charles Clevenger**

USA

**Hiram Cody**

USA

**Kelly Coffey**

UK

**Mark Cohen**

USA

**Bertrand Coiffier**

France

**Gisele Colleoni**

Brazil

**Marco Colleoni**

Italy

**Luis Colomo**

Spain

**Emile Comans**

Netherlands

**Niamh Conlon**

USA

**Nathan Consedine**

New Zealand

**Alfredo Conti**

Italy

**William Omar Contreras Lopez**

Colombia

**Giovanni Conzo**

Italy

**Darrel Cook**

Canada

**Kumarasen Cooper**

USA

**Wendy Cooper**

Australia

**Rob Coppes**

Netherlands

**Pierre Cordelier**

France

**Seth Joel Corey**

USA

**Giacomo Corrado**

Italy

**Mark Corrigan**

Ireland

**Javier Cortes Bordoy**

Spain

**Giuliana Cortese**

Italy

**Laura Cortesi**

Italy

**Renzo Corvo**

Italy

**Silvia Liliana Cossio**

Brazil

**Frederico Costa**

Brazil

**Jose Luis Costa**

Portugal

**Vera Costa**

Portugal

**Massimo Costantini**

Italy

**Gregory Cote**

USA

**Paul Cottu**

France

**Fergus Couch**

USA

**David Countryman**

USA

**Johannes Coy**

Germany

**Susanna Cramb**

Australia

**Ross Cranston**

USA

**S. Michael Crawford**

UK

**Sara Cresta**

Italy

**Carmen Criscitiello**

Italy

**Cinzia Crivellaro**

Italy

**Alysha Croker**

Canada

**Colleen Croniger**

USA

**Tim Crook**

UK

**Nina Crowley**

USA

**Ofelia Cruz**

Spain

**Vincent Cryns**

USA

**Joaquin Cubiella**

Spain

**Alessandro Cucchetti**

Italy

**Ioan Cucoranu**

USA

**Ziyou Cui**

USA

**S. Nicole Culos-Reed**

Canada

**Jeff Cummings**

UK

**Antonio Cuneo**

Italy

**Margaret Currie**

New Zealand

**Karen Curtin**

USA

**Kate Cuschieri**

UK

**Vahid Reza Dabbagh Kakhki**

Iran

**Gabi U Dachs**

New Zealand

**Alv A. Dahl**

Norway

**Liping Dai**

USA

**Adrien Daigeler**

Germany

**Maria Dalamaga**

Greece

**Katiuscia Dallaglio**

Italy

**Christopher Daly**

USA

**Nadia Dandachi**

Austria

**Mark Danese**

USA

**Cyril Danjoux**

Canada

**Silvia Darb-Esfahani**

Germany

**Özlem Darcansoy Iseri**

Turkey

**Kakoli Das**

Singapore

**Mukul Das**

India

**Kaustubh Datta**

USA

**Heena Dave**

USA

**Ben Davidson**

Norway

**Guido Davidzon**

USA

**Elizabeth Davies**

UK

**Jean-Luc Davignon**

France

**Sanford Dawsey**

USA

**Heather Dawson**

Switzerland

**Jonathan Day**

USA

**Prithwish De**

Canada

**Francesca De Amicis**

Italy

**Michele De Bortoli**

Italy

**Filippo De Braud**

Italy

**Patricia De Cremoux**

France

**Pierre De Delva**

USA

**Sonia De Francesco**

Italy

**Valli De Re**

Italy

**Silvia De Sanjose**

Spain

**Ines De Santiago**

UK

**Paulo De Sepulveda**

France

**Marco De Velasco**

Japan

**Henry John Christiaan De Vries**

Netherlands

**Olivier De Wever**

Belgium

**Alberto Deganello**

Italy

**Tim Dekker**

Netherlands

**Mario Del Rosso**

Italy

**Michael Dellinger**

USA

**Wendy Demark-Wahnefried**

USA

**Metin Demirkaya**

Turkey

**Semra Demokan**

Turkey

**An-Mei Deng**

China

**Dajun Deng**

China

**Zhao-Qun Deng**

China

**Carsten Denkert**

Germany

**Lynette Denny**

South Africa

**Susan Dent**

Canada

**Elena Deryugina**

USA

**Anjali Deshpande**

USA

**Beena Cr Devi**

Malaysia

**Sarah Dewilde**

Belgium

**Nandini Dey**

USA

**Subhojit Dey**

India

**Saravana Dhanasekaran**

USA

**Debanjan Dhar**

USA

**Santosh Dhule**

USA

**Pietro Di Fazio**

Germany

**Giuseppe Di Lorenzo**

Italy

**Massimo Di Maio**

Italy

**Luca Di Tommaso**

Italy

**Eleftherios Diamandis**

Canada

**Sérgio Dias**

Portugal

**Stefan Diem**

Switzerland

**Veronique Dieras**

France

**Jill Dietz**

USA

**Analisa Difeo**

USA

**Ekkehard Dikomey**

Germany

**Mary Dillhoff**

USA

**Gabriel Dimattia**

Canada

**Johannes Carl Athanasios Dimopoulos**

Greece

**Goberdhan Dimri**

USA

**Pei-Rong Ding**

China

**Wen-Xing Ding**

USA

**Lijun Ding**

China

**Marina Diniz**

Brazil

**Andreas Dinkel**

Germany

**Peter Dipasco**

USA

**Bahar Dirican**

Turkey

**Kristin Dittmar**

USA

**Thomas Dittmar**

Germany

**Francesco Dituri**

Italy

**Leo Ditzel Filho**

USA

**Tayyab Diwan**

USA

**Michael Dixon**

UK

**Hooman Djaladat**

USA

**Mehdi Djekidel**

USA

**Khanh Do**

USA

**Alexander Dobrovic**

Australia

**Epameinondas Dogeas**

USA

**Jennifer Doherty**

USA

**Ramona Dolscheid-Pommerich**

Germany

**Gemma Dominguez**

Spain

**Davide Maria Donati**

Italy

**Nicholas Donato**

USA

**Jia-Hong Dong**

China

**Qihan Dong**

Australia

**Xiaojing Dong**

China

**Yuan Dongmei**

China

**Joseph Dort**

Canada

**C George Priya Doss**

India

**Isabel Dos Santos Silva**

UK

**Maura Dowling**

Ireland

**Amy Downing**

UK

**Jerome Doyen**

France

**Tommaso Dragani**

Italy

**Richard Drake**

USA

**Didier Dreau**

USA

**Paul Drew**

Australia

**Gypsyamber D'Souza**

USA

**Jiajun Du**

China

**Haifeng Duan**

China

**Shiwei Duan**

China

**Tao (Tony) Duan**

China

**Shikha Dubey**

India

**Anna Dubrovska**

Germany

**Francois Ducray**

France

**Vikas Dudeja**

USA

**Maire Duggan**

Canada

**Pierre-Antoine Dugué**

Denmark

**Lucien Duijm**

Netherlands

**Martine Dunnwald**

USA

**Francine Durocher**

Canada

**Adriana Dusso**

Spain

**Janice Dutcher**

USA

**Amit Dutt**

India

**Roisin Dwyer**

Ireland

**Lars Dyrskjøt**

Denmark

**Piotr Dziegiel**

Poland

**Peter Dziegielewski**

USA

**Alan Eastman**

USA

**Jeanette Eckel-Passow**

USA

**Iris Eder**

Austria

**Anders Edsjö**

Sweden

**Dylan Edwards**

UK

**Joanne Edwards**

UK

**Mary Egan**

Canada

**Marcelo Ehrlich**

Israel

**Pieter Eichhorn**

Singapore

**Günter Eisele**

Switzerland

**Wolfgang Eisterer**

Austria

**Ram Eitan**

Israel

**Lars Ekblad**

Sweden

**Fathia El Sharkawi**

Egypt

**Samer El-Dika**

USA

**Olivier Elemento**

USA

**Daniel Elias**

Ethiopia

**Rossella Elisei**

Italy

**Lesley Ellies**

USA

**Jacqueline Ellis**

UK

**Jean Anderson Eloy**

USA

**J. Mark Elwood**

New Zealand

**Cynthia Emory**

USA

**Agamemnon Epenetos**

UK

**Lisa Ercolano**

USA

**Costantino Errani**

Italy

**Cam Escoffery**

USA

**Hannu Eskola**

Finland

**Jose M. Estrela**

Spain

**Arnold Etame**

USA

**Joseph Evans**

USA

**Matthias Evert**

Germany

**Eduardo Eyras**

Spain

**Antonio Facchiano**

Italy

**Johannes Fahrmann**

USA

**Stefano Fais**

Italy

**Jean Faivre**

France

**Marwan Fakih**

USA

**Zhining Fan**

China

**Jia Fan**

China

**Lifang Fan**

China

**Meiyun Fan**

USA

**Xing Fan**

USA

**Bingliang Fang**

USA

**Fu-Min Fang**

Taiwan

**Qingming Fang**

USA

**Xiao Fang**

China

**Jia Fang**

USA

**Xiaoguang Fang**

USA

**Eduardo Farias**

USA

**Lucyana Farias**

Brazil

**Mohamad Farid**

Singapore

**Ahmad Faried**

Japan

**Fabio Farinati**

Italy

**Daniel Farkas**

USA

**Rosa Farràs**

Spain

**Peter Fasching**

Germany

**Ambrogio Fassina**

Italy

**Elvira Favoino**

Italy

**Jerome Fayette**

France

**Veronika Fedirko**

USA

**Manuel Felices**

Sweden

**Jorge Felix**

Portugal

**Richard Feltbower**

UK

**Angelo Pietro Femia**

Italy

**Nicola Fenderico**

Italy

**Annika Fendler**

Germany

**Hanping Feng**

USA

**Lynnette Robin Ferguson**

New Zealand

**Almudena Fernandez Briera**

Spain

**Arias Fernando**

Spain

**Mårten Fernö**

Sweden

**Cristina Ferrás**

Portugal

**Irene Ferrer**

Spain

**Yonatan Feuermann**

Germany

**Mary Jo Fidler**

USA

**Ryan Fields**

USA

**Joana Figueiredo**

Portugal

**Jorge Filmus**

Canada

**Jeffrey Fine**

USA

**Daniel Fink**

Switzerland

**Gary Firestone**

USA

**Jodie M Fleming**

USA

**Emmy Fleuren**

Netherlands

**Mirembe Florenc**

Uganda

**Tullio Florio**

Italy

**Uta Flucke**

Netherlands

**Emmanouil Fokas**

Germany

**Maria Aparecida A Koike Folgueira**

Brazil

**Marco Folini**

Italy

**Gunnar Folprecht**

Germany

**Kam Weng Fong**

Singapore

**Laia Font**

Spain

**Caterina Fontanella**

Germany

**Lindsay Forbes**

UK

**Marvella Ford**

USA

**Charles Forscher**

USA

**Paul Foster**

UK

**Julie Fradette**

Canada

**Silvia Franceschi**

France

**Valerie Francescutti**

Canada

**Renato Franco**

Italy

**Philippe Frank**

USA

**Callum Fraser**

UK

**Jonna Frasor**

USA

**Alex Freeman**

UK

**Manuel Fresno**

Spain

**Daniela Frezzetti**

Italy

**Rafael Fridman**

USA

**Philip Friedlander**

USA

**Daniel Frigo**

USA

**Jan Frisell**

Sweden

**Holger Fröhlich**

Germany

**Olivia Fromigue**

France

**Michael Frumovitz**

USA

**Shen Fu**

China

**Loning Fu**

USA

**Siqing Fu**

USA

**Xianghui Fu**

USA

**Xiao-Long Fu**

China

**Fumihiko Fujita**

Japan

**Kazuya Fukuoka**

Japan

**Ada Funaro**

Italy

**Pauline Funchain**

USA

**Yusuke Furukawa**

Japan

**Junji Furuse**

Japan

**Koh Furuta**

Japan

**Mayuko Furuta**

Japan

**Hideki Furuya**

USA

**Martin Götte**

Germany

**Shiva Keshava Gaddam B**

USA

**Jochen Gaedcke**

Germany

**Bertino Gaetano**

Italy

**Ajeet Gajra**

USA

**Umberto Galderisi**

Italy

**Miguel Gallego**

Spain

**Brenda Gallie**

Canada

**Valerio Gallotta**

Italy

**Paolo Gandellini**

Italy

**Ripal Gandhi**

USA

**Sara Gandini**

Italy

**Wei-Qiang Gao**

China

**Ai Gao**

China

**Chunfang Gao**

China

**Qinyan Gao**

China

**Paul Gao**

USA

**Haidong Gao**

China

**Wen Gao**

China

**Claus Garbe**

Germany

**Spiros D. Garbis**

UK

**Juan F Garcia**

Spain

**Montse Garcia**

Spain

**José Manuel García-Heredia**

Spain

**Ramón García-Sanz**

Spain

**Paul Gardner**

USA

**David Garfield**

China

**Manuela Gariboldi**

Italy

**Scott Garrett**

USA

**Gasim Gasim**

Sudan

**Pierluigi Gasparini**

USA

**Mia Gaudet**

USA

**Luke Gaughan**

UK

**Julie Gavard**

France

**Maria Gavriatopoulou**

Greece

**Hiram Gay**

USA

**Bishoy Gayed**

USA

**Christopher Gayer**

USA

**Akihiko Gemma**

Japan

**Domenico Genovesi**

Italy

**Alex Georgakilas**

Greece

**Vassilis Georgoulias**

Greece

**Holger Gerullis**

Germany

**Robert Getzenberg**

USA

**Sara Ghaderi**

Norway

**Farid Ghadessy**

Singapore

**Hazem Ghebeh Saudi**

Arabia

**Paola Ghiorzo**

Italy

**Paramita Ghosh**

USA

**Goutam Ghosh Choudhury**

USA

**Juliana Giacomazzi**

Brazil

**Vincenzo Giancotti**

Italy

**Elisa Giannoni**

Italy

**Bruce Giantonio**

USA

**Spencer Gibson**

Canada

**Roben Gieling**

UK

**Gretchen Gierach**

USA

**Carolina Gigek**

Brazil

**Ignacio Gil-Bazo**

Spain

**Ethel Gilbert**

USA

**Blake Gilks**

Canada

**Jerome Gilleron**

Germany

**Bernhard Gillissen**

Germany

**Etel Gimba**

Brazil

**Antonio Z Gimeno García**

Spain

**Doron Ginsberg**

Israel

**Rosaria Giordano**

Italy

**Paolo Giorgi Rossi**

Italy

**Elisa Giovannetti**

Netherlands

**Albert Girotti**

USA

**David Gius**

USA

**Babak Givi**

USA

**Simona Glasberg**

Israel

**David Glass**

USA

**Sacha Gnjatic**

USA

**Roseline Godbout**

Canada

**James Goedert**

USA

**James Going**

UK

**Vita Golubovskaya**

USA

**Carolina Gomes**

Brazil

**Dawidson Gomes**

Brazil

**Jorge Gomez**

USA

**Isabel Gomez Monterrey**

Italy

**Anne Gompel**

France

**Elena Goncharova**

USA

**Zhaohui Gong**

China

**Céline Gongora**

France

**Lou Gonsalves**

USA

**Maria Cristina Gonzalez**

Brazil

**Patrick Gonzalez**

France

**Patrick Goodman**

Ireland

**Satish Gopal**

Malawi

**Priya Gopalan**

USA

**Gregor Gorkiewicz**

Austria

**Rei Goto**

Japan

**Michael Gottesman**

USA

**Ioannis Gounaris**

UK

**Erin Grady**

USA

**Ullrich Graeven**

Germany

**Maria Gramatges**

USA

**Diana Greenfield**

UK

**Darren C Greenwood**

UK

**Peter Greer**

Canada

**Christopher Griffith**

USA

**Thomas Griffith**

USA

**Giovanni Grignani**

Italy

**Anita Grigoriadis**

UK

**Christoph Grimm**

Austria

**Chloe Grimmett**

UK

**Peter Grimminger**

Germany

**Fabio Grizzi**

Italy

**Christian Grohe**

Germany

**Francesco Grossi**

Italy

**Kenneth Grossmann**

USA

**Beth Grunfeld**

UK

**Eva Grunfeld**

Canada

**Viktor Grünwald**

Germany

**Guoli Gu**

China

**Yajun Gu**

China

**Xiaoxiang Guan**

China

**Yongsong Guan**

China

**Nathalie Guedj**

France

**Arndt Guentsch**

Germany

**Vince Guerriero**

USA

**Jih-Hwa Guh**

Taiwan

**Massimo Guidoboni**

Italy

**Maria Valle Guijarro**

USA

**Andre Guimaraes**

Brazil

**Margaret Gulley**

USA

**Mehmet Gunduz**

Japan

**Deliang Guo**

USA

**Wei Guo**

China

**Li Guo**

China

**Wei-Jian Guo**

China

**Xiaozhong Guo**

China

**Zong Sheng Guo**

USA

**Junming Guo**

China

**Digant Gupta**

USA

**Nalini Gupta**

India

**Phalguni Gupta**

India

**Marianne Guren**

Norway

**Kurt Gust**

USA

**Dmitriy Gutkin**

USA

**Mina Ha**

South Korea

**Ulrich Hacker**

Germany

**Christina Hackl**

Germany

**Ahmed Haddad**

USA

**Peyman Hadji**

Germany

**Costas Hadjipanayis**

USA

**Andreas Hadjisavvas**

Cyprus

**Anne Irene Hagen**

Norway

**Ki-Baik Hahm**

South Korea

**Benjamin Haibe-Kains**

Canada

**Jörg Haier**

Germany

**Hidde Haisma**

Netherlands

**A Ari Hakimi**

USA

**Richard Halberg**

USA

**Michael Halpern**

USA

**Gerhard Hamilton**

Austria

**William Hamilton**

UK

**Bruce Hammock**

USA

**Weidong Han**

China

**Wenling Han**

China

**Henry Han**

USA

**Yong Han**

China

**Rachel Hanisch**

France

**Matthew Hankins**

UK

**Torben Hansen**

Denmark

**Dapeng Hao**

Macao

**Jihui Hao**

China

**Sheng-Po Hao**

Taiwan

**Hisashi Harada**

USA

**Jennifer Hardingham**

Australia

**James Hardwick**

Netherlands

**Ljubica Harhaji**

Serbia

**Valsala Haridas**

USA

**J. Chuck Harrell**

USA

**Holly Harris**

USA

**Steven Hart**

USA

**Lowell Hart**

USA

**Jaana Hartikainen**

Finland

**John Hartley**

UK

**Sheri Hartman**

USA

**Sayed Hashemi**

Netherlands

**Naozumi Hashimoto**

Japan

**Noor Hasima**

Malaysia

**Brian Hasinoff**

Canada

**Ursula Hasler-Strub**

Switzerland

**Cesare Hassan**

Italy

**Raffit Hassan**

USA

**Brad Haverkos**

USA

**Lukas J.A.C. Hawinkels**

Netherlands

**David Hawkins**

USA

**Sandi Hayes**

Australia

**John Hays**

USA

**Aiwu He**

USA

**Hong He**

Australia

**Jin He**

USA

**Qing He**

China

**Shannon Healy**

Canada

**Lionel Hebbard**

Australia

**Ingrid Hedenfalk**

Sweden

**Steffen Heeg**

Germany

**Christopher Heeschen**

Spain

**Balazs Hegedus**

Austria

**Axel Hegele**

Germany

**Joost Hegmans**

Netherlands

**Tuomas Heikkinen**

Finland

**Max Heiland**

Germany

**Elmar Heinrich**

Germany

**Florian Heitz**

Germany

**Ellen Heitzer**

Austria

**Eric Henderson**

USA

**Mary Hendrix**

USA

**Charles Hennemeyer**

USA

**John Henson**

USA

**Javier Hernandez-Losa**

Spain

**Shawn Hervey-Jumper**

USA

**Marcin Hetnal**

Poland

**Michael Heuser**

Germany

**Holger Heyn**

Spain

**Aram Hezel**

USA

**Toyoaki Hida**

Japan

**Jin-Ichi Hida**

Japan

**Lotte Hiddingh**

Netherlands

**Geoff Higgins**

UK

**Mitsunori Higuchi**

Japan

**Masahiro Higuchi**

USA

**Frans Hilgers**

Netherlands

**Andrew Hill**

New Zealand

**Uwe Himmelreich**

Belgium

**Joe Hines**

USA

**Okio Hino**

Japan

**Christoph Hirche**

Germany

**Seiichi Hirota**

Japan

**Bradford Hirsch**

USA

**Hal Hirte**

Canada

**Asahi Hishida**

Japan

**Taro Hitosugi**

USA

**Steven Hochwald**

USA

**Robert Hoffman**

USA

**Michael Hoffmeister**

Germany

**Ilse Hofmann**

Germany

**Thomas Hofmann**

Germany

**Peter Hollenhorst**

USA

**Antoinette Hollestelle**

Netherlands

**Lars Holmberg**

UK

**Dennis Holmes**

USA

**Jeff Holst**

Australia

**Santosh Honavar**

India

**Kazuo Honda**

Japan

**Angela Hong**

Australia

**Chi-Chen Hong**

USA

**Yong Sang Hong**

South Korea

**Kimi Honma**

Australia

**Naoko Honma**

Japan

**Craig Horbinski**

USA

**Ryouichi Horie**

Japan

**Hidehito Horinouchi**

Japan

**Martinho Horta**

Brazil

**H Dean Hosgood**

USA

**Isamu Hoshino**

Japan

**Katsuyuki Hotta**

Japan

**Scott Howard**

USA

**Debra Howell**

UK

**Viive Howell**

Australia

**Morten Høyer**

Denmark

**Jenn-Ren Hsiao**

Taiwan

**Michael Hsiao**

Taiwan

**Chiun Hsu**

Taiwan

**Cheng-Lung Hsu**

Taiwan

**Brian Hu**

USA

**Chaosu Hu**

China

**Yi Hu**

China

**Xi Chun Hu**

China

**Hsuan-Ying Huang**

Taiwan

**Cai Huang**

USA

**Chih-Jen Huang**

Taiwan

**Chi-Cheng Huang**

Taiwan

**Chao-Cheng Huang**

Taiwan

**Eng-Yen Huang**

Taiwan

**Wen-Yi Huang**

USA

**Ying Huang**

USA

**Yi Huang**

USA

**Zhaohui Huang**

China

**Weihuang Huang**

China

**Paul Huang**

UK

**Michael Hubalek**

Austria

**Peter Huber**

Germany

**John Hudson**

Canada

**David Hughes**

USA

**Jooryung Huh**

South Korea

**Stefanie Huhn**

Germany

**David Hui**

USA

**Zhouguang Hui**

China

**Luo Huiyan**

China

**Michael Hundemer**

Germany

**Erik Huntzicker**

USA

**Sara Hurvitz**

USA

**Jayne Hutchinson**

UK

**Ji-Young Hwang**

South Korea

**Tae-Ho Hwang**

South Korea

**Elias Hyams**

USA

**Per Hydbring**

USA

**David Hyman**

USA

**Nancy Hynes**

Switzerland

**Ichinosuke Hyodo**

Japan

**Roberto Iacovelli**

Italy

**Merdol Ibrahim**

UK

**Wataru Ichikawa**

Japan

**Kenneth Iczkowski**

USA

**Satoshi Igawa**

Japan

**Michaela Ihle**

Germany

**Hiroshi Ikai**

Japan

**Junichiro Ikeda**

Japan

**Masahide Ikeguchi**

Japan

**Carolina Ilkow**

Canada

**Martin Illemann**

Denmark

**Mohammad Ilyas**

UK

**Kazushi Imai**

Japan

**Takeshi Imamura**

Japan

**Stefano Indraccolo**

Italy

**Maurizio V Infante**

Italy

**Robert Ingham**

Canada

**Pasquale Innominato**

France

**Hidenori Inohara**

Japan

**Akira Inoue**

Japan

**Paul Insel**

USA

**Marisa Ionta**

Brazil

**Marilena Iorio**

Italy

**Sarosh Irani**

UK

**Elena Irollo**

Germany

**Claudio Isella**

Italy

**Ciro Isidoro**

Italy

**Nina Isoherranen**

USA

**Yasuhiro Ito**

Japan

**Yuri Ito**

Japan

**Ken Itoh**

Japan

**Motoki Iwasaki**

Japan

**Yoshimi Iwasaki**

Japan

**Tomoko Iwata**

UK

**Obiajulu Iwenofu**

USA

**N Gopalakrishna Iyer**

Singapore

**Jakob Izbicki**

Germany

**Evgeny Izumchenko**

USA

**Maria Jackson**

Jamaica

**Anne-Birgitte Jacobsen**

Norway

**Judith S Jacobson**

USA

**Marie-Lise Jaffrain-Rea**

Italy

**Harriët Jager-Wittenaar**

Netherlands

**Preetesh Jain**

USA

**Praveen Jaiswal**

India

**Jens Jakob**

Germany

**Marko Jakopovic**

Croatia

**Sirpa Jalkanen**

Finland

**Kaiser Jamil**

India

**Sabina Janciauskiene**

Sweden

**Tae Jung Jang**

South Korea

**Judith Jans**

Netherlands

**Emiel Janssen**

Norway

**Klaus-Peter Janssen**

Germany

**Thierry Jarde**

Australia

**Mona Jeffreys**

UK

**Valerie Jenkins**

UK

**Jørgen Bjerggaard Jensen**

Denmark

**Roxanne Jensen**

USA

**Hae Myung Jeon**

South Korea

**Seong-Yun Jeong**

South Korea

**Branislav Jeremic**

Serbia

**Fredrik Jerhammar**

Sweden

**Carmen Jeronimo**

Portugal

**Udo Jeschke**

Germany

**Mahsa Jessri**

Canada

**John Jessup**

USA

**Hernandez Jesus M**

Spain

**Collene Jeter**

USA

**Bruno Jham**

USA

**Guixiang Ji**

China

**Xin-Ying Ji**

China

**Weiping Jia**

China

**Wei Jiang**

China

**Min Jiang**

China

**Wen Jiang**

USA

**Shih Sheng Jiang**

Taiwan

**Tun Jie**

USA

**Tang Jie**

China

**Camilo Jimenez**

USA

**Rodrigo Jimenez Garcia**

Spain

**Haifeng Jin**

China

**Shouguang Jin**

USA

**Young Woo Jin**

South Korea

**Chunxia Jing**

China

**Young Suk Jo**

South Korea

**Aart G Jochemsen**

Netherlands

**Karin Joehrer**

Austria

**Markus Joerger**

Switzerland

**David Johnson**

USA

**Matthew Johnson**

USA

**Theron Johnson**

Germany

**Mickaila Johnston**

USA

**V. Craig Jordan**

USA

**Bharat Joshi**

USA

**Elmar Joura**

Austria

**Alicja Jozkowicz**

Poland

**Manfred Jücker**

Germany

**Ian Judson**

UK

**Bettina Julin**

Sweden

**Andreas Jung**

Germany

**Young Jung**

South Korea

**Kerstin Junker**

Germany

**Therese Juul**

Denmark

**Guillermo Juvenal**

Argentina

**Hayyam Kiratli**

Turkey

**Lars Kaderali**

Germany

**Pinar Kadioglu**

Turkey

**Shigenori Kadowaki**

Japan

**Keita Kai**

Japan

**Sham Kakar**

USA

**Kiran Kakarala**

USA

**John Kalbfleisch**

USA

**Vijaykumar Kale**

USA

**Kevin Kalinsky**

USA

**Enikoe Kallay**

Austria

**Galatea Kallergi**

Greece

**Raghu Kalluri**

USA

**Charis Kalogirou**

Germany

**Christina Kalpadakis**

Greece

**Arif Kamal**

USA

**Bozena Kaminska**

Poland

**Izumi Kamiya**

Japan

**Ellen Kampman**

Netherlands

**Ghodsieh Kamrani**

Iran

**Ryuichi Kanai**

Japan

**Kent Kanao**

Japan

**Mustapha Kandouz**

USA

**Kazunori Kanehira**

USA

**Peggy Kanellou**

Greece

**Jung Hun Kang**

South Korea

**Stephan Kanzler**

Germany

**Jessica Kao**

USA

**Matthias Kappler**

Germany

**Georgios Karagkounis**

USA

**Amer Karam**

USA

**Jose Karam**

USA

**Anna Karlsson**

Sweden

**Rotem Karni**

Israel

**Radha Krishna Murthy Karuturi**

Singapore

**Takahiro Kasamatsu**

Japan

**Stefan Kasper**

Germany

**Wassim Kassouf**

Canada

**Kota Katanoda**

Japan

**Katsuaki Kato**

Japan

**Masaru Katoh**

Japan

**Rachel Katzenellenbogen**

USA

**Rondi Kauffmann**

USA

**Ruchika Kaul-Ghanekar**

India

**Koji Kawakami**

Japan

**Chul-Seung Kay**

South Korea

**Youqiang Ke**

UK

**Bhumsuk Keam**

South Korea

**Ulrich Keilholz**

Germany

**Linda Kelemen**

Canada

**Kimberly Kelly**

USA

**Patrick Kelly**

USA

**Stefan Kempa**

Germany

**Paraic Kenny**

USA

**Lucyna Kepka**

Poland

**Ruth Keri**

USA

**Michael Kershaw**

Australia

**Santosh Kesari**

USA

**Aparna Kesarwala**

USA

**Dineo Khabele**

USA

**Hadi Khan**

USA

**Kum Kum Khanna**

Australia

**Adnan Khattak**

Australia

**Seong Lin Khaw**

Australia

**Surapan Khunamornpong**

Thailand

**Tobias Kiesslich**

Austria

**Thomas Kietzmann**

Finland

**Kotaro Kiga**

Japan

**Sarah Kiguli**

Uganda

**Eiji Kikuchi**

Japan

**Elaine Kilgour**

UK

**Hyoung-Ryoul Kim**

South Korea

**Eugene Kim**

USA

**Hyung-Ho Kim**

South Korea

**Jung-Ae Kim**

South Korea

**James Kim**

USA

**Joseph Kim**

USA

**Betty Y.S. Kim**

USA

**Su Woon Kim**

Singapore

**Sung Joo Kim**

South Korea

**Marianne Kim**

USA

**Sun Jung Kim**

South Korea

**Tae Won Kim**

South Korea

**Won Gu Kim**

South Korea

**Young Wan Kim**

South Korea

**T Peter Kingham**

USA

**Iva Kirac**

Croatia

**Jutta Kirfel**

Germany

**Hiroyuki Kirikoshi**

Japan

**Yohei Kirino**

USA

**Antti Kivelä**

Finland

**Kristina Kjaerheim**

Norway

**Wolfram Klapper**

Germany

**Giseli Klassen**

Brazil

**Christoph Klein**

Germany

**Martin Klein**

Netherlands

**Robert Klein**

USA

**Miriam Kleiter**

Austria

**Samuel Klempner**

USA

**Dennis Klinman**

USA

**Roman Kloeckner**

Germany

**Petra Klose**

Germany

**Olaf Klungel**

Netherlands

**Stian Knappskog**

Norway

**Michael Knauer**

Switzerland

**Steen Knudsen**

Denmark

**Ben Ko**

Hong Kong

**Jiunn-Liang Ko**

Taiwan

**Michiya Kobayashi**

Japan

**Wayne Koch**

USA

**Hemant Kocher**

UK

**Martin Koebel**

Canada

**Devin Koestler**

USA

**Barbara Kofler**

Austria

**Fumitaka Koga**

Japan

**Takahiko Kogai**

Japan

**Yasuhiro Koh**

Japan

**Takashi Kohno**

Japan

**Yasuo Kokai**

Japan

**Anna Kolovou**

Greece

**Hiroshi Kondoh**

Japan

**Konstantinos Konstantopoulos**

USA

**Christos Kontos**

Greece

**Despina Kontos**

USA

**Rituraj Konwar**

India

**József Kónya**

Hungary

**Bon-Kyoung Koo**

UK

**Dong Hoe Koo**

South Korea

**Joseph Koopmeiners**

USA

**Marta Korbonits**

Uruguay

**Murray Korc**

USA

**Hasan Korkaya**

USA

**Penelope Korkolopoulou**

Greece

**Masafumi Koshiyama**

Japan

**Zsofia Kote-Jarai**

UK

**Madhuri Koti**

Canada

**Ashwin Kotnis**

India

**Svetlana Kotova**

USA

**Athanasios Kotsakis**

Greece

**Athanassios Kotsinas**

Greece

**Vassilis Kouloulias**

Greece

**Zaklina Kovacevic**

Australia

**Gyoergy Kovacs**

Germany

**Nobuyuki Koyama**

Japan

**Sergei Kozlov**

Australia

**Shingo Kozono**

Japan

**Dominique Kranz**

Germany

**Pawel Krawczyk**

Poland

**Pavel Krejci**

Czech Republic

**Marina Kreutz**

Germany

**Samaya Krishnan**

USA

**Lasse Sommer Kristensen**

Denmark

**Stephan Kruck**

Germany

**Wlodzimierz Krzyzosiak**

Poland

**Rui Kuang**

USA

**Lisha Kuang**

USA

**Lucia Kucerova**

Slovakia

**Veerapol Kukongviriyapan**

Thailand

**Teru Kumagi**

Japan

**Ashok Kumar**

USA

**Shiyam Kumar**

Oman

**Prince Kumar**

India

**Ranjit Kumar**

USA

**Gopal Kundu**

India

**Hsiu-Ni Kung**

Taiwan

**Etsuo Kunieda**

Japan

**Joachim Kunz**

Germany

**Catarina Alisa Kunze**

Germany

**Jolanta Kupryjanczyk**

Poland

**Kentaro Kurashina**

Japan

**Masafumi Kurosumi**

Japan

**Boris Kuvshinoff**

USA

**Hiroyuki Kuwano**

Japan

**Marilyn Kwan**

USA

**Ioannis Kyvernitakis**

Germany

**Simon Laban**

Germany

**Chann Lagadec**

France

**Andrei Lagaru**

UK

**Catherine Lai**

USA

**Lily Lai**

USA

**Barry Laird**

UK

**Lisenn Lalier**

France

**Vaia Lambadiari**

Greece

**Daniel Lambert**

UK

**Nadia Lampiasi**

Italy

**Jeffrey Landercasper**

USA

**Deanna Lane**

USA

**Kerstin Lang**

Germany

**Sven Lang**

Germany

**Rupert Langer**

Switzerland

**Cord Langner**

Austria

**Andrea Lania**

Italy

**Helena Länsimies-Antikainen**

Finland

**Constantin Lapa**

Germany

**Michael Larkin**

UK

**Mette Bach Larsen**

Denmark

**Christer Larsson**

Sweden

**Ahmed Lasfar**

USA

**Ulrik Lassen**

Denmark

**Justin Lathia**

USA

**David Latini**

USA

**Joseph W.Y. Lau**

Hong Kong

**Darryl Lau**

USA

**Vincent Launay-Vacher**

France

**Matthias Lauth**

Germany

**Nicole Lavender**

USA

**Yuri Lazebnik**

USA

**Lu Le**

USA

**Avi Leader**

Israel

**Sophie Lebel**

Canada

**Arier Lee**

New Zealand

**Cheng Chi Lee**

USA

**Cheng-Han Lee**

Canada

**Chien-Hung Lee**

Taiwan

**Hsin-Chen Lee**

Taiwan

**Ching-Chih Lee**

Taiwan

**Han-Woong Lee**

South Korea

**Hyeon-Woo Lee**

South Korea

**Jae Lyun Lee**

South Korea

**Jung-Il Lee**

South Korea

**Ju-Seog Lee**

USA

**Jehn Chuan Lee**

Taiwan

**Hwajeong Lee**

USA

**Jung-Min Lee**

USA

**Kin Chung Lee**

Hong Kong

**Se-Hoon Lee**

South Korea

**Simon J. Craddock Lee**

USA

**Kin Wah Lee**

Hong Kong

**Wei-Hwa Lee**

Taiwan

**Qunying Lei**

China

**Mario Leitao Jr**

USA

**Catherine Lejeune**

France

**Sophie Lelievre**

USA

**Wei-Dong Leng**

China

**Claudia Lengerke**

Switzerland

**Jochen Lennerz**

USA

**Ryan Lennon**

USA

**Dido Lenze**

Germany

**Francesco Leone**

Italy

**Derek Leroith**

USA

**Gregory Lesinski**

USA

**Eleonora Leucci**

Belgium

**Anna Leung**

USA

**David Levens**

USA

**Francis Levi**

France

**Baoqing Li**

USA

**Chunyu Li**

China

**Jin Li**

China

**Huabing Li**

USA

**Jingmei Li**

Singapore

**Shau-Hsuan Li**

Taiwan

**Wei Li**

USA

**Hongyan Li**

USA

**Jinjun Li**

China

**Long-Cheng Li**

USA

**Shao-Qiang Li**

China

**Xiaobo Li**

China

**Jianming Li**

China

**Jun Li**

China

**Long Shan Li**

USA

**Madeline Li**

Canada

**Qiang Li**

USA

**Rui Li**

China

**Wen Li**

China

**Ya-Hsin Li**

Taiwan

**Zaibo Li**

USA

**Min Lian**

USA

**Evi Lianidou**

Greece

**Yung-Po Liaw**

Taiwan

**Massimo Libra**

Italy

**Karen Liby**

USA

**Jonathan Licht**

USA

**David Liebner**

USA

**Sandra Liekens**

Belgium

**Jong-Baeck Lim**

South Korea

**Carmen Lima**

Brazil

**Carlos A. Lima**

Brazil

**Chih-Lung Lin**

Taiwan

**Ho-Sheng Lin**

USA

**Huey-Jen Lin**

USA

**Jiumao Lin**

China

**Kenneth Lin**

USA

**Kwang-Huei Lin**

Taiwan

**Yu-Sheng Lin**

USA

**Li Lin**

China

**Lijuan Lin**

China

**Wei-Che Lin**

Taiwan

**Xun Lin**

USA

**Zhenhua Lin**

China

**Hannah Linden**

USA

**Chen Ling**

USA

**Zhi-Qiang Ling**

China

**Isaac Lipkus**

USA

**Baorui Liu**

China

**Hong Liu**

USA

**Delong Liu**

USA

**Ji-Bin Liu**

USA

**Lifang Liu**

Netherlands

**Houbao Liu**

China

**Tianshu Liu**

China

**Bo Liu**

China

**Jianming Liu**

Cocos Keeling Islands

**Zuqiang Liu**

USA

**Shing-Hwa Liu**

Taiwan

**Tao Liu**

Australia

**Weiguo Liu**

USA

**Xiangguo Liu**

China

**Xiaochun Liu**

USA

**Yan Liu**

USA

**Zhili Liu**

China

**Zhensheng Liu**

USA

**Gema Llort**

Spain

**Bryan Lo**

Canada

**Simon Lo**

USA

**Filippo Lococo**

Italy

**Ines Lohse**

Canada

**Niklas Loman**

Sweden

**Giuseppe Lombardi**

Italy

**Fulvio Lonardo**

USA

**Cheryl London**

USA

**Kara Long Roche**

USA

**Adhemar Longatto Filho**

Portugal

**Luca Longo**

Italy

**Elena Lopez**

Spain

**Gonzalo Lopez**

USA

**Jose Antonio Lopez-Guerrero**

Spain

**Francisco Lopez-Hernandez**

Spain

**Ian Lorimer**

Canada

**Attila Lorincz**

UK

**Joannie Lortet-Tieulent**

USA

**Corina Lorz**

Spain

**Kevin R. Loughlin**

USA

**Laercio Gomes Lourenco**

Brazil

**Aoife Lowery**

Ireland

**Hua Lu**

USA

**Kai-Hsi Lu**

Taiwan

**Shen Lu**

China

**Xiaomei Lu**

China

**Weiyue Lu**

China

**Sudjit Luanpitpong**

USA

**Giovanni Lughezzani**

Italy

**Alessandro Lugli**

Switzerland

**Eiliv Lund**

Norway

**Kenneth Lundstrom**

Switzerland

**Pengcheng Luo**

China

**Sheng-Dean Luo**

Taiwan

**Shuhong Luo**

USA

**Yunus Luqmani**

Kuwait

**John Lurain**

USA

**Rodney Luwor**

Australia

**Yi Lv**

China

**Herbert Lyerly**

USA

**Courtney Lyles**

USA

**Marita Lynagh**

Australia

**Heidi Lyng**

Norway

**Hong Ma**

China

**Chenming Ma**

Germany

**Daoxin Ma**

China

**Jun Ma**

China

**Lin Ma**

China

**Qingyong Ma**

China

**Bruce Macher**

USA

**Gerardo Mackenzie**

USA

**Angeliki Magklara**

Greece

**Kelly Magliocca**

USA

**Jamal Mahajna**

Israel

**Marthandan Mahalingam**

USA

**Umesh Mahanshetty**

India

**Reza Mahdavi**

Iran

**Georg Mahlknecht**

Israel

**Mamun Mahtab**

Bangladesh

**Hai-Qiang Mai**

China

**Shishir Maithel**

USA

**Shahana Majid**

USA

**Keivan Majidzadeh-A**

Iran

**Carl Maki**

USA

**Paolo Malatesta**

Italy

**Núria Malats**

Spain

**Jitka Malcikova**

Czech Republic

**Peter Malfertheiner**

Germany

**Eirik Malinen**

Norway

**Sandra Mallone**

Italy

**Joshua Mammen**

USA

**Aaron Mammoser**

USA

**Kwan Man**

China

**Parmeet Manchanda**

USA

**Julien Mancini**

France

**Chandi Mandal**

India

**Syamsundar Mandal**

India

**Yefim Manevich**

USA

**Sylvain Manfredi**

France

**Sendurai Mani**

USA

**David Mankoff**

USA

**Melissa Mannion**

USA

**Ulrich Mansmann**

Germany

**Li Mao**

USA

**Xinliang Mao**

China

**Christophe Kamungu Mapendano**

Denmark

**Jodi Maranchie**

USA

**Paola Marcato**

Canada

**Federica Marchesi**

Italy

**Julian Marchesi**

UK

**Sergio Marchini**

Italy

**Caterina Marchiò**

Italy

**Raphaël Maréchal**

Belgium

**Johanna Maree**

South Africa

**Julie Margenthaler**

USA

**Boris Margulis Russian**

Federation

**Vitaly Margulis**

USA

**Isabelle Maridonneau-Parini**

France

**Jose Marin**

Spain

**Adrian Mariño-Enriquez**

USA

**Talar Markossian**

USA

**Athina Markou**

Greece

**Janina Markowska**

Poland

**David Marples**

UK

**Iván Márquez-Rodas**

Spain

**Fatima Martel**

Portugal

**Janet Martin**

Australia

**Robert Martin**

USA

**Javier Martin-Broto**

Spain

**Juan Martinez**

Spain

**Claudia Martini**

Italy

**Kouji Maruyama**

Japan

**Atsushi Masamune**

Japan

**Floriana Mascilini**

Italy

**Liliane Massade**

France

**Andrew Massey**

UK

**Norikazu Masuda**

Japan

**María-Victoria Mateos**

Spain

**Augusto Matos**

Portugal

**Ayako Matsuda**

Japan

**Sachiko Matsuhashi**

Japan

**Ken Matsui**

USA

**Kazumasa Matsumoto**

Japan

**Shingo Matsumoto**

Japan

**Yukinori Matsuo**

Japan

**Hideyasu Matsuyama**

Japan

**Matthias Matter**

Switzerland

**Ines Mattos**

Brazil

**Estella Matutes**

Spain

**Loredana Mauro**

Italy

**Felicity E B May**

UK

**Philippa May**

UK

**Joel Mayerson**

USA

**Eleni Mayson**

Australia

**Pawel Mazur**

USA

**Guillermo Mazzolini**

Argentina

**Sam Mbulaiteye**

USA

**Florencia McAllister**

USA

**Shelli McAlpine**

Australia

**Pamela McCall**

UK

**Georgia McCann**

USA

**Damian McCartan**

Ireland

**Anne Marie McCarthy**

USA

**James A McCaul**

UK

**Sara McComb**

USA

**Carmel McConville**

UK

**Paula McDonald**

UK

**Paul McDonald**

Canada

**Braden McFarland**

USA

**Ronald McGarry**

USA

**Mark McKeage**

New Zealand

**Margaret McLaughlin-Drubin**

USA

**Philip McLoone**

UK

**Megan McNally**

USA

**Mairead McNamara**

Canada

**Stephen McPherson**

Australia

**Amy McQueen**

USA

**Justin McWilliams**

USA

**Alan Meeker**

USA

**Michal Mego**

USA

**Gabor Mehes**

Hungary

**Rohit Mehra**

USA

**Shikhar Mehrotra**

USA

**Davide Melisi**

Italy

**Anders Mellemgaard**

Denmark

**Lighua Meng**

China

**Li Rong Meng**

Macao

**Maxwell Meng**

USA

**Wei Meng**

China

**Lourdes Mengual**

Spain

**Kavitha Menon**

New Zealand

**Rolf Mentlein**

Germany

**Andres Merits**

Estonia

**Marco Carlo Merlano**

Italy

**Douglas Scott Merrell**

USA

**Megan Merrill**

USA

**Wilma Mesker**

Netherlands

**Giuseppe Alessio Messano**

Italy

**Giulio Metro**

Italy

**Delia Mezzanzanica**

Italy

**Francesca Micci**

Norway

**George Michalopoulos**

USA

**Gaetane Michaud**

USA

**Pellegrino Michele**

Italy

**Mark Middleton**

UK

**James Mier**

USA

**Antimo Migliaccio**

Italy

**Lucia Migliore**

Italy

**Atsushi Miki**

Japan

**Wolfgang Mikulits**

Austria

**Branko Miladinovic**

USA

**Michele Milella**

Italy

**David Miles**

UK

**Donald Miller**

USA

**William Miller**

UK

**Frederick Millham**

USA

**Abul Milton**

Australia

**Seigo Minami**

Japan

**Marek Minarik**

USA

**Christoph Minichsdorfer**

Austria

**Pamela Minicozzi**

Italy

**Cindy Miranti**

USA

**Sameer Mirza**

USA

**Shunichi Misawa**

Japan

**Anjali Mishra**

India

**Dhruva Kumar Mishra**

USA

**Anand Kumar Mishra**

India

**Shiraz Mishra**

USA

**Akira Mitsuhashi**

Japan

**Makito Miyake**

Japan

**Hiroshi Miyamoto**

USA

**Akihiko Miyanaga**

Japan

**Yoshihiro Miyasaka**

Japan

**Minoru Miyashita**

Japan

**Yoshihiko Miyata**

Japan

**Keiko Miyoshi**

Japan

**Xianming Mo**

China

**Helmout Modjtahedi**

UK

**Markus Moehler**

Germany

**Cathy Moelans**

Netherlands

**Siver Andreas Moestue**

Norway

**Maddalena Mognato**

Italy

**Mohammad Mohammadianpanah**

Iran

**Altaf Mohammed**

USA

**Daniela Molena**

USA

**Alessio Molfino**

Italy

**Manuel Molina**

USA

**Sonia Molina-Pinelo**

Spain

**Francesca Molinari**

Switzerland

**Pål Møller**

Norway

**Chiara Mondello**

Italy

**Bradley Monk**

USA

**Ali Montazeri**

Iran

**Filippo Montemurro**

Italy

**Corey Montgomery**

USA

**Rodolfo Montironi**

Italy

**Dan Moore**

USA

**Roger Moorehead**

Canada

**Martin Mørck Mortensen**

Denmark

**James Morden**

UK

**Carlos Moreno**

USA

**Victor Moreno**

Spain

**Eiichi Morii**

Japan

**Toshikazu Moriwaki**

Japan

**Laura Moro**

Italy

**Denis Moro-Sibilot**

France

**David Morris**

Australia

**Joanna Moschandreas**

Greece

**Joachim Mössner**

Germany

**Aldo Mottino**

Argentina

**Marcella Mottolese**

Italy

**Giannis Mountzios**

France

**Mirna Mourtada-Maarabouni**

UK

**Mohamed Moustafa**

Lebanon

**Marek Mraz**

Czech Republic

**Ewa Mrozek**

USA

**David Mu**

USA

**Colin Muirhead**

UK

**Ken-Ichi Mukaisho**

Japan

**Arnab Mukherjea**

USA

**Malini Mukherjee**

USA

**Yoshiki Mukudai**

Japan

**Catherine Muller**

France

**Cecile Muller**

Australia

**Patricia Muller**

UK

**Sandra Muñoz-Galván**

Spain

**Alastair Munro**

UK

**Jordi Muntané**

Spain

**Takashi Murakami**

Japan

**Raul Murillo**

Colombia

**Mandi Murph**

USA

**Jamie Murphy**

UK

**William Murphy**

USA

**Graeme Murray**

UK

**Douglas Murrey**

USA

**Charles Murtaugh**

USA

**Muhammed Murtaza**

USA

**Teemu Murtola**

Finland

**Lucia Anna Muscarella**

Jordan

**Peter Muscarella**

USA

**Anna Maria Musti**

Italy

**Domenica Musumeci**

Italy

**Rajaram Nagarajan**

USA

**Pratik Nagaria**

USA

**Clara Nahmias**

France

**Shan Naidu**

USA

**Norifumi Naka**

Japan

**Mitsutoshi Nakada**

Japan

**Ikuhiko Nakase**

Japan

**Tetsuya Nakatsura**

Japan

**Tomio Nakayama**

Japan

**Harikrishna Nakshatri**

USA

**Joo-Hyun Nam**

South Korea

**Nisana Namwat**

Thailand

**Marius Nap**

Netherlands

**Gopeshwar Narayan**

USA

**Steven Narod**

Canada

**Tatiana Nasedkina**

Russian Federation

**Rachael Natrajan**

UK

**Shoji Natsugoe**

Japan

**Inna Naumov**

Israel

**Pierina Navarria**

Italy

**Alfons Navarro**

Spain

**Silvio Naviglio**

Italy

**Lakshmi Nayak**

Uganda

**Richard Neal**

UK

**Theo Nell South**

Africa

**Erik Nelson**

USA

**Camilla Nero**

Italy

**Jochen Neuhaus**

Germany

**Jens Neumann**

Germany

**Bart Neyns**

Belgium

**Jennifer Nicholas**

UK

**Anthony Nichols**

Canada

**Simone Niclou**

Luxembourg

**Alcina Nicol**

Brazil

**Carlos Nicolau**

Spain

**Francesco Nicolini**

UK

**Anders Lade Nielsen**

Denmark

**Finn Cilius Nielsen**

Denmark

**Torsten Nielsen**

Canada

**Giuseppe Nigri**

Italy

**Yuzuru Niibe**

Japan

**Hiroaki Niiro**

Japan

**Jonas Nilsson**

Sweden

**Mikael Nilsson**

Sweden

**Katharina Nimptsch**

USA

**Noriyuki Nishimura**

USA

**Tomohiro Nishina**

Japan

**Benjamin Nisman**

Israel

**Xiaohui Niu**

China

**Na Niu**

USA

**Sisse Helle Njor**

Denmark

**Makoto Noda**

Japan

**Véronique Noé**

Spain

**Lucien Noens**

Belgium

**Maria Claudia Nogueira Zerbini**

Brazil

**Dong-Young Noh**

South Korea

**Sung Hoon Noh**

South Korea

**Nijiro Nohata**

USA

**Takashi Nomiyama**

Japan

**Helena Nordenstedt**

Sweden

**Nicola Normanno**

Italy

**Daniel Nowak**

Germany

**Ula Nur**

UK

**Mari Nygård**

Norway

**Irena Oblak**

Slovenia

**Dan O'Connell**

Canada

**James O'Connor**

UK

**Ina Oehme**

Germany

**Anthonia Ogbera**

Nigeria

**Shuji Ogino**

USA

**Daniel Oh**

USA

**Robert Ohgami**

USA

**Kouki Ohtsuka**

Japan

**Akinyemi Ojesina**

USA

**Yusuke Oji**

Japan

**Hidenori Ojima**

Japan

**Hirohisa Okabe**

Japan

**Kinya Okamoto**

Japan

**Alicia Okines**

UK

**Natsuko Okita**

Japan

**Yusuke Okuma**

Japan

**Lajos Olasz**

Hungary

**Raymond Oliphant**

UK

**Carla Oliveira**

Portugal

**Cristina Oliveira**

Brazil

**Leticia Oliveira-Ferrer**

Germany

**Lisa Oliver**

France

**Micheau Olivier**

France

**Jordan Olson**

USA

**Robert Olson**

Canada

**Ian Olver**

Australia

**David Omalley**

USA

**Suzanne O'Neill**

USA

**Jason Ong**

Australia

**Evan Ong**

USA

**Hiroshi Onishi**

Japan

**Maurizio Onisto**

Italy

**Betul Oran**

USA

**Pedro Orihuela**

Chile

**Josep Oriola**

Spain

**Rosaria Orlandi**

Italy

**Daniel Orringer**

USA

**Richard Orton**

UK

**Mitsuhiko Osaki**

Japan

**Daniel O'Shannessy**

USA

**Sergej P. Osinsky**

Ukraine

**Marie Stampe Ostenfeld**

Denmark

**Silvia Ottaviani**

UK

**Leo Otterbein**

USA

**Penelope Ottewell**

UK

**Laura Ottini**

Italy

**Mototsugu Oya**

Japan

**Agostino Paccagnella**

Italy

**Leisl Packer**

Australia

**Roman Paduch**

Poland

**Gabriella Pagnan**

Italy

**Katia Pagnano**

Brazil

**Young-Ki Paik**

South Korea

**Tuya Pal**

USA

**Pierlorenzo Pallante**

Italy

**Athanasios Pallis**

Greece

**Trenis Palmer**

USA

**Marta Palmieri**

Italy

**Moritz Palmowski**

Germany

**Grazia Palomba**

Italy

**Jianji Pan**

China

**Jingxuan Pan**

China

**Sai Yi Pan**

Canada

**Katherine Panageas**

USA

**Vijay Pandey**

Singapore

**Gopi Krishna Panicker**

India

**Carolina Panis**

Brazil

**Kostas Pantopoulos**

Canada

**Alexandros Papalambros**

Greece

**George Papaxoinis**

Greece

**Laura Papi**

Italy

**Emmanouil Pappou**

USA

**Angelo Paradiso**

Italy

**Bhupesh Parashar**

USA

**Christos Paraskeva**

UK

**Olivier Pardo**

UK

**Rahul Parikh**

USA

**Carol Parise**

USA

**Changwon Park**

USA

**Hee Chul Park**

South Korea

**Jinha Park**

USA

**Seho Park**

South Korea

**Kyu-Sang Park**

South Korea

**Sook Ryun Park**

South Korea

**Maurizio Parola**

Italy

**Paola Parrella**

Italy

**Luca Parrillo**

Italy

**Toshima Parris**

Sweden

**Barbara Parsons**

USA

**Valeria Pascale**

Italy

**Steve Pascolo**

Germany

**Eddy Pasquier**

Australia

**Marilo Pastor**

Spain

**Sandra Pastorino**

USA

**Roberta Pastorino**

Italy

**Asmita Patel**

New Zealand

**Jitesh Patel**

USA

**Maitraya Patel**

USA

**Prabhudas Patel**

India

**Nitin Patil**

Germany

**Deepa Patil**

USA

**Vijay Patil**

India

**Ruth Patterson**

USA

**Nicholas Pavlidis**

Greece

**Laura Pazzaglia**

Italy

**Jing Pei**

China

**Helene Pelicano**

USA

**Jerry Pelletier**

Canada

**Juha Peltonen**

Finland

**Du Peng**

China

**Fangyu Peng**

USA

**Xiaohua Peng**

USA

**Jun Peng**

China

**Marzia Pennati**

Italy

**Núbia Braga Pereira**

Brazil

**Francesco Perrone**

Italy

**Federica Perrone**

Italy

**Luca Persano**

Italy

**Manuel Perucho**

USA

**Janette Perz**

Australia

**Patrick Petignat**

Switzerland

**Stefano Petti**

Italy

**Vincenzo Pezzi**

Italy

**Claudia Pföhler**

Germany

**Prakash Babu Phanithi**

India

**Lynette Phillips**

USA

**Gary Piazza**

USA

**Giulia Piazzi**

Italy

**Maria Carmela Piccirillo**

Italy

**Alain Piche**

Canada

**Martin Pichler**

Austria

**Phillip Pierorazio**

USA

**Pages Pierre-Benoit**

France

**Maria Catherine Pietanza**

USA

**Thierry Pieters**

Belgium

**Filippo Pietrantonio**

Italy

**Melissa Pilewskie**

USA

**Radhakrishna Pillai**

India

**Dietmar Pils**

Austria

**Pascal Pineau**

France

**Benjamin Pineda**

Mexico

**Céline Pinheiro**

Brazil

**Maria Simona Pino**

Italy

**Ruben Pio**

Spain

**Grzegorz Piotrowski**

Poland

**Martin Pirkl**

Germany

**Jose Piruat**

Spain

**Salvatore Piscuoglio**

USA

**Vincenzo Pitini**

Italy

**Sara Pizzamiglio**

Italy

**Laura Pizzuti**

Italy

**Andrzej Plawski**

Poland

**Ruben Plentz**

Germany

**Miklos Pless**

Switzerland

**Ferdinand Ploner**

Austria

**Janice Pluth**

USA

**Martin Pölcher**

Germany

**Hans-Christian Pommergaard**

Denmark

**Mathurose Ponglikitmongkol**

Thailand

**Brenten Popiel**

USA

**Helena Pópulo**

Portugal

**Luca Porcu**

Italy

**Paola Postacchini**

Italy

**David Potter**

USA

**George Poultsides**

USA

**Philippe Pourquier**

France

**Ned Powell**

UK

**Emily Power**

UK

**Samantha Pozzi**

Italy

**C Pramesh**

India

**Sandip Prasad**

USA

**Aleix Prat**

Spain

**Sarah Preisler**

Denmark

**Hans Prenen**

Belgium

**Ana Preto**

Portugal

**Allan Price**

UK

**Shawn Price**

USA

**Timothy Price**

Australia

**Ivanka Prichard**

Australia

**Alessandro Prinetti**

Italy

**Andrea Prodosmo**

Italy

**Sandi Pruitt**

USA

**Giancarlo Pruneri**

Italy

**Christopher Przybycin**

USA

**Dimitri Psimaras**

France

**Ashok Pullikuth**

USA

**Marco Pupo**

Italy

**Michele Purrello**

Italy

**Brigitte Pützer**

Germany

**Wei-Xiang Qi**

China

**Chengyong Qin**

China

**Xue Qin**

China

**Jingxin Qiu**

USA

**Xiujuan Qu**

China

**Marcelo Queiroz**

Switzerland

**Richard Quek**

Singapore

**Hilmar Quentmeier**

Germany

**Victor Quesada**

Spain

**Peter Quesenberry**

USA

**Andrew Quest**

Chile

**Patrick Quint**

USA

**Jürgen Radons**

Germany

**Milan Radovich**

USA

**Colin Rae**

UK

**Sajjad Rafiq**

UK

**Nuh Rahbari**

Germany

**Francesca Raimondo**

Italy

**Michal Rajski**

Switzerland

**Janusz Rak**

Canada

**Stephen Ramsey**

USA

**Guglielmina Ranzani**

Italy

**Ganesh Rao**

USA

**Rajini Rao**

USA

**Kati Rasanen**

Finland

**Dominique Rash**

USA

**Timothy Ratliff**

USA

**Elena Ratner**

USA

**Edward Ratovitski**

USA

**Tilman Rau**

Switzerland

**Paul Rava**

USA

**Pritha Ray**

India

**Karine Raymond**

France

**Matejka Rebolj**

Denmark

**Diana Redwood**

USA

**Katherine Reeves**

USA

**David Regan**

Australia

**Enrique Regidor**

Spain

**Alistair Reid**

UK

**Anne Reiman**

UK

**Hong Ren**

China

**Noa Rensing**

USA

**Diana Resendez**

Mexico

**Fabien Reyal**

France

**Carolina Reyes**

USA

**Jordan Reynolds**

USA

**Domenico Ribatti**

Italy

**Carmela Ricciardelli**

Australia

**Jeremy Rich**

USA

**Holger Richly**

Germany

**Christopher Ricketts**

USA

**Johannes Rieger**

Germany

**Piotr Rieske**

Poland

**Jonathan Riess**

USA

**Oliver Riesterer**

Switzerland

**Michel Rigaud**

France

**Guido Rindi**

Italy

**Michael Risk**

USA

**Susan Rittling**

USA

**Juan Carklos Roa**

Chile

**Karin Roberg**

Sweden

**Jacques Robert**

France

**Ryan Roberts**

USA

**Keith Robertson**

USA

**Kim Robien**

USA

**Cliff Robinson**

USA

**Trude Eid Robsahm**

Norway

**Mark Robson**

USA

**Marco Rocchi**

Italy

**Sonia Rocha**

UK

**Franz Rödel**

Germany

**Rb Roden**

USA

**Wendy Rodenburg**

Netherlands

**Mauricio Rodriguez Dorantes**

Mexico

**Socorro Maria Rodriguez Pinilla**

Spain

**Claire Rodriguez-Lafrasse**

France

**Achim Rody**

Germany

**Falk Roeder**

Germany

**Bernard Roelen**

Netherlands

**Sebastian Rogenhofer**

Germany

**Simon Rogers**

UK

**Stephen Rohan**

USA

**Christian Rolfo**

Belgium

**Maurizio Romano**

Italy

**Margarita Romeo**

Spain

**Chiara Romualdi**

Italy

**Minhua Rong**

China

**Dana Roque**

USA

**Antonia Roseweir**

UK

**Antonella Rosi**

Italy

**Stephanie Rosse**

USA

**Charles Rosser**

USA

**Sabrina Rossi**

Jordan

**Matteo Rota**

Italy

**Johann Rotheneder**

Austria

**Etienne Rouleau**

France

**Jason Roy**

USA

**Shreyas Roy**

USA

**Susanta Roychoudhury**

India

**Giovina Ruberti**

Italy

**Luca Rubino**

Italy

**Randall Ruch**

USA

**Volker Rudat Saudi**

Arabia

**Christian Rudlowski**

Germany

**Emil Rudolf**

Czech Republic

**Anja Rudolph**

Germany

**Montserrat Rue**

Spain

**Claudia Ruebe**

Germany

**Corina S. Rueegg**

Switzerland

**Thomas Ruhstaller**

Switzerland

**Alvaro Ruibal**

Spain

**Roslin Russell**

UK

**Martin Ruthardt**

Germany

**Jim Rutka**

Canada

**Munindra Ruwali**

India

**Lisa Rydén**

Sweden

**Sepideh Saadatmand**

Netherlands

**Kauko Samuel Saarilahti**

Finland

**Renaud Sabatier**

France

**Vera Saddi**

Brazil

**Andrea Sadlonova**

USA

**Toshiaki Saeki**

Japan

**Carmen Saez**

Spain

**Motoyasu Sagawa**

Japan

**Manujendra Saha**

Canada

**Solmaz Sahebjam**

USA

**Abdul Saied**

USA

**Neeru Saini**

India

**Yoshifumi Saisho**

Japan

**Hiroshi Saito**

Japan

**Kazutaka Saito**

Japan

**Yuki Saito**

Japan

**Masao Saitoh**

Japan

**Eiji Sakai**

Japan

**Yasunaru Sakuma**

Japan

**Ritu Salani**

USA

**Dolores Salas**

Spain

**Mauricio Salcedo**

Mexico

**Saima Saleem**

Pakistan

**Nikolaos Salemis**

Greece

**David Saltman**

Canada

**Vanda Salutari**

Italy

**Ignacio San Francisco**

Chile

**Margarita Sanchez-Beato**

Spain

**Rupninder Sandhu**

USA

**Juan Sandoval**

Spain

**Sumathi Sankaran-Walters**

USA

**Sampath Santhosh**

India

**Marta Santisteban**

Spain

**Matteo Santoni**

Italy

**Eric Santoni-Rugiu**

Denmark

**Sankar Sanyal**

India

**Gabriele Saretzki**

UK

**Luis Sarian**

Brazil

**Sulen Sarioglu**

Turkey

**Balazs Sarkadi**

Hungary

**Ana Bela Sarmento-Ribeiro**

Portugal

**Pablo Sarobe**

Spain

**Aaron Sarver**

USA

**Shizuka Sasazuki**

Japan

**Takami Sato**

USA

**Taroh Satoh**

Japan

**Vishnupriya Satti**

India

**Torill Sauer**

Norway

**Niramol Savaraj**

USA

**Norie Sawada**

Japan

**Okay Saydam**

Austria

**Bruna Scaggiante**

Italy

**Aldo Scarpa**

Italy

**Karin Scarpinato**

USA

**Michael Schaapveld**

Netherlands

**Beat Schäfer**

Switzerland

**Florence Schaffner**

France

**Helmut Schaider**

Australia

**Catherine Schairer**

USA

**Marilyn Schapira**

USA

**Tom Scharschmidt**

USA

**Laura Scherer**

USA

**Roxana Schillaci**

Argentina

**Christoph Schlapbach**

Switzerland

**Paul G Schlegel**

Germany

**Martina Schmidt**

Netherlands

**Fernando Schmitt**

Portugal

**Wolfgang Schmitt**

Germany

**Marc Schmitz**

Germany

**Paul Magnus Schneider**

Switzerland

**Birgit Schoeberl**

USA

**Markus Scholz**

Germany

**Mario Schootman**

USA

**Andres Jan Schrader**

Germany

**Michael Georg Schrauder**

Germany

**Michael Schuler**

Germany

**Linda Schuler**

USA

**Wolfgang Schulz**

Germany

**Udo Schumacher**

Germany

**Eric Schuur**

USA

**Gerrit Schuurhuis**

Netherlands

**Thomas Schwaab**

USA

**Lee Schwartzberg**

USA

**Heidi Schwarzenbach**

Germany

**Lukas Schwentner**

Germany

**Kathryn Schwertfeger**

USA

**Andreas Scorilas**

Greece

**Suzanne Scott**

UK

**Anna Ivana Scovassi**

Italy

**Beatrice Seddon**

UK

**Carmen Segrelles Huelga**

Spain

**Masahiro Seike**

Japan

**Takahiro Seki**

Sweden

**Michaela Semeraro**

France

**Bo Kyoung Seo**

South Korea

**Yeon Sun Seong**

South Korea

**Massimo Serra**

Italy

**Mª Jose Serrano**

Spain

**Rosario Serrano**

Spain

**Raquel Seruca**

Portugal

**Gautam Sethi**

USA

**Bhuvana Setty**

USA

**Patricia Severino**

Brazil

**Alex Sgambato**

Italy

**Mehdi Shakibaei**

Germany

**Ali Shamseddine**

Lebanon

**Xiying Shang**

USA

**Jieya Shao**

USA

**Anna Shapiro**

USA

**Shashwat Sharad**

USA

**Akash Sharma**

USA

**Dipali Sharma**

USA

**Linda Sharp**

Ireland

**Leslie Shaw**

USA

**Richard Shaw**

UK

**Paul Shaw**

UK

**Kieran Sheahan**

Italy

**Jason Sheehan**

USA

**Bairong Shen**

China

**Rulong Shen**

USA

**Yaxing Shen**

China

**Lizong Shen**

China

**Ke Sheng**

USA

**Elizabeth Shephard**

UK

**Trevor Shepherd**

Canada

**Kirti Shetty**

USA

**Runhua Shi**

USA

**Ruihua Shi**

China

**Ming Shi**

China

**Hisayuki Shigematsu**

Japan

**Jin-Yuan Shih**

Taiwan

**Naoto Shikama**

Japan

**Takeo Shimasaki**

Japan

**Shoji Shimoyama**

Japan

**Myung-Geun Shin**

South Korea

**Hai-Rim Shin**

Philippines

**Yoshiyuki Shioyama**

Japan

**Hidenori Shiraha**

Japan

**Kohei Shitara**

Japan

**Yow-Ling Shiue**

Taiwan

**Farbod Shojaei**

USA

**Hirokazu Shoji**

Japan

**Lynette Sholl**

USA

**Noam Shomron**

Israel

**Susan Short**

UK

**Mohamed Shoukri**

Saudi Arabia

**Samia Shouman**

Egypt

**Viji Shridhar**

USA

**Stuti Shroff**

USA

**Ting Si**

China

**Kenneth Siddle**

UK

**Lucas Sideris**

Canada

**Anna Sidorchuk**

Sweden

**Gene Siegal**

USA

**Robert Siegel**

Germany

**Cornelis Sier**

Netherlands

**Luca Sigalotti**

Italy

**Ana Elizabete Silva**

Brazil

**Paulo Silveira**

Brazil

**Marko Simunovic**

Canada

**Alessandro Sindoni**

Italy

**Amanpal Singh**

USA

**Pankaj Singh**

USA

**Praby Singh**

USA

**Rakesh Singh**

USA

**Sheila Singh**

Canada

**Aatur Singhi**

USA

**Prem Sinha**

USA

**Birandra Sinha**

USA

**Ronit Sionov**

Israel

**Alphonse Sirica**

USA

**Diego Sisci**

Italy

**Shankar Siva**

Australia

**Tobias Sjöblom**

Sweden

**James Skipworth**

UK

**Frank Slack**

USA

**Andrzej Slominski**

USA

**Peter Sminia**

Netherlands

**Alexandra Smith**

UK

**Bryan Smith**

USA

**Gary Smith**

USA

**Steven Smith**

USA

**Peter Smyth**

Ireland

**Asley Snider**

USA

**Jonathan Soboloff**

USA

**Silvia Soddu**

Italy

**Elin Söderberg**

Sweden

**Artem Sokolov**

USA

**Maria Soldatkina**

Ukraine

**Xavier Sole**

Spain

**Ponnandai Somasundar**

USA

**Alvaro Somoza**

Spain

**Yong Song**

China

**Yong Sang Song**

South Korea

**Guru Sonpavde**

USA

**Richie Soong**

Singapore

**Prasanna Sooriakumaran**

UK

**Halfdan Sorbye**

Norway

**Karina Dalsgaard Sorensen**

Denmark

**Irma Soria-Mercado**

Mexico

**José Luis Soto**

Spain

**John Souglakos**

Greece

**Harmik Soukiasian**

USA

**Paul Span**

Netherlands

**Valerie Speirs**

UK

**Claudio Spick**

Austria

**Gian Paolo Spinelli**

Italy

**Gilbert Spizzo**

Italy

**Jeremy Squire**

Brazil

**Nahida Srabovic**

Bosnia and Herzegovina

**Sriganesh Srihari**

India

**Sanjay K. Srivastava**

USA

**Elvira Stacher**

Austria

**John Staffurth**

UK

**John Stagg**

Canada

**Venturina Stagni**

Italy

**Olle Stål**

Sweden

**Helge Stalsberg**

Norway

**Daniel Starczynowski**

USA

**Andreas Stark**

Germany

**Kristen Stashek**

USA

**Giorgio Stassi**

Italy

**Georgios Stathopoulos**

Greece

**Rafal Stec**

Poland

**Sandra Steffens**

Germany

**Guenther Steger**

Austria

**Eva Steliarova-Foucher**

France

**Ulf-Hakan Stenman**

Finland

**Nathan Stephens**

UK

**Richard Stevens**

USA

**Christopher Stevenson**

Australia

**Anton Stift**

Austria

**Charles Stiller**

UK

**Christopher Stipp**

USA

**Lucia Anna Stivala**

Italy

**Andrew Stoker**

UK

**Katja Storch**

Germany

**Douglas Strand**

USA

**Carina Strell**

Sweden

**Michael Stroehlein**

Germany

**Fay Strohschein**

Canada

**Darrin Stuart**

USA

**Dominik Sturm**

Germany

**Chun-Li Su**

Taiwan

**Rongjian Su**

China

**Changqing Su**

China

**Jin Su**

China

**Li-Jen Su**

Taiwan

**Subbaya Subramanian**

USA

**Takeshi Suda**

Japan

**Tamotsu Sugai**

Japan

**Tomoharu Sugie**

Japan

**Mitsushige Sugimoto**

Japan

**Haruhiko Sugimura**

Japan

**Kevin Sun**

New Zealand

**Shi-Yong Sun**

USA

**Jing Sun**

China

**Yihua Sun**

China

**Shaogang Sun**

USA

**Xinchen Sun**

China

**Yanan Sun**

China

**Wei Sun**

China

**Wenxiang Sun**

China

**Yu Sunakawa**

USA

**Debasish Sundi**

USA

**Joseph Sung**

Hong Kong

**Zhenhe Suo**

Norway

**Rajah Supramaniam**

Australia

**Alexey Surov**

Germany

**Larry Suva**

USA

**Akihiko Suzuki**

Japan

**Eiichiro Suzuki**

Japan

**Hidekazu Suzuki**

Japan

**Yutaka Suzuki**

Japan

**Rajaraman Swaminathan**

India

**Benjamin Swanson**

USA

**Colleen Sweeney**

USA

**Fred Sweep**

Netherlands

**Peter Szabo**

USA

**Marta Szajnik**

Poland

**Tibor Szarvas**

Austria

**Peter Szlosarek**

UK

**Krzysztof (Chris) Szyfter**

Poland

**Yoshiaki Tabuchi**

Japan

**Michael Tachezy**

Germany

**Yuji Tachimori**

Japan

**Keiichiro Tada**

Japan

**Elda Tagliabue**

Italy

**Pierosandro Tagliaferri**

Italy

**Alberto Tagliafico**

Italy

**Ayumu Taguchi**

USA

**Sk Tai**

Taiwan

**Daisuke Takahari**

Japan

**Ryuji Takahashi**

Japan

**Atsuo Takashima**

Japan

**Ken-Ichi Takayama**

Japan

**Tetsuo Takehara**

Japan

**Akinobu Taketomi**

Japan

**Kengo Takeuchi**

Japan

**Jun-Ichi Tamaru**

Japan

**Daniel Tan**

Singapore

**Eng Huat Tan**

Singapore

**Hiromi Tanaka**

USA

**Motoyoshi Tanaka**

Japan

**Cristiana Tanase**

Romania

**Kari Tanderup**

USA

**Naoko Tanese**

USA

**Dabei Tang**

China

**Hua Tang**

China

**Xinyu Tang**

USA

**Robert M. Tanguay**

Canada

**Philippe Taniere**

UK

**Pieter Tanis**

Netherlands

**Edward Tanner**

USA

**Tamara Tanos**

Italy

**Hideki Tanzawa**

Japan

**Lei Tao**

China

**Raquel Tarazona**

Spain

**Jussi Tarkkanen**

Finland

**Line Tarpgaard**

Denmark

**Natasha Tasevska**

USA

**Tyna Taskila**

UK

**Mine Islimye Taskin**

USA

**Pierfrancesco Tassone**

Italy

**Helge Taubert**

Germany

**Sun Kuie Tay**

Singapore

**Sally Taylor**

UK

**Georgi Tchernev**

Bulgaria

**Muy-Kheng M. Tea**

Austria

**Rajeshwar Rao Tekmal**

USA

**Teruhiko Terasawa**

Japan

**Evangelos Terpos**

Greece

**Luigi Terracciano**

Switzerland

**Mary Beth Terry**

USA

**Paul Terry**

USA

**Giovanni Tesoriere**

Italy

**Anna Tessari**

USA

**Krishnansu Tewari**

USA

**George Thalmann**

Switzerland

**Douglas Thamm**

USA

**Ekkasit Tharavichitkul**

Thailand

**Kullathorn Thephamongkhol**

Thailand

**Brigitte Thériault**

Canada

**Anne Thiebaut**

France

**Susan Thomas**

USA

**Linda Thompson**

USA

**John Thoms**

Canada

**Mathilde Borg Houlberg Thomsen**

Denmark

**Steven Thorpe**

USA

**Vesteinn Thorsson**

USA

**Nirav Thosani**

USA

**Inger Thune**

Norway

**Peter Thuss-Patience**

Germany

**Bharat Thyagarajan**

USA

**Ivana Ticha**

Czech Republic

**Shweta Tikoo**

Australia

**David Timson**

UK

**Andrea Tinelli**

Italy

**Ingeborg Tinhofer**

Germany

**Shinsaku Togo**

Japan

**Chee-Keong Toh**

Singapore

**Yuji Toiyama**

Japan

**Arinobu Tojo**

Japan

**Rodrigo Toledo**

USA

**Nori Tolosa De Talamoni**

Argentina

**Federica Tomao**

Italy

**Marco Tomasetti**

Italy

**Yoshihiro Tominaga**

Japan

**Patricia Tonin**

Canada

**Jyoti Tope**

India

**Matthew Topham**

USA

**Erkan Topkan**

Turkey

**Paul Toren**

Canada

**Toshihiko Torigoe**

Japan

**Luigi Tornillo**

Switzerland

**Anna Torres**

Poland

**Alicia Torriglia**

France

**Suzy Torti**

USA

**Yann Touchefeu**

France

**Marina Touillaud**

France

**Amanda Townsend**

Australia

**Nham Tran**

Australia

**Olivier Tredan**

France

**Marjolijn Trietsch**

Netherlands

**Fabio Trippa**

Italy

**Tiziana Triulzi**

Italy

**Lucio Trodella**

Italy

**Volodymyr Tryndyak**

USA

**Kenneth Tsai**

USA

**Sen-Tien Tsai**

Taiwan

**Nadejda Tsankova**

USA

**George Sai-Wah Tsao**

Hong Kong

**Vassiliki Liana Tsikitis**

USA

**Konstantinos Tsilidis**

Greece

**George Tsirakis**

Greece

**Kelvin Tsoi**

Hong Kong

**Tung Yu Tsui**

Germany

**Takemasa Tsuji**

USA

**Ryouichi Tsunedomi**

Japan

**Nelson Hirokazu Tsuno**

Japan

**Jan Tuckermann**

Germany

**Musaffe Tuna**

USA

**Yusuf Tutar**

USA

**James Tysome**

UK

**George Tzanakakis**

Greece

**Flora Tzelepis**

Australia

**Keisuke Uehara**

Japan

**Selma Ugurel**

Germany

**Eugen Uhlmann**

Germany

**Nancy Uhrhammer**

France

**Shigeki Umemura**

Japan

**Gerold Untergasser**

Austria

**Susanne Unverzagt**

Germany

**Ghanshyam Upadhyay**

India

**Cheerag Upadhyaya**

USA

**Jean-Luc Urbain**

USA

**Fyodor Urnov**

USA

**Ruchan Uslu**

Turkey

**Jaydutt Vadgama**

USA

**Ratna Vadlamudi**

USA

**Jayant Vaidya**

UK

**Antonis Valachis**

Greece

**Guido Valente**

Italy

**Daniel Vallbohmer**

Germany

**Sonia Vallet**

Germany

**Lampros Vamvakas**

Greece

**Charles Van Berlo**

Netherlands

**Elske Van Den Akker**

Netherlands

**Jasper G. Van Den Boorn**

Germany

**Donald L. Van Der Peet**

Netherlands

**W.A. Van Der Zwan**

Netherlands

**Carolien Van Deurzen**

Netherlands

**Carla Van Gils**

Netherlands

**Elke Van Hoof**

Belgium

**Leonie Van Hulsteijn**

Netherlands

**Marlous Van Laar**

UK

**Ryan Van Laar**

USA

**Marieke Van Leeuwen**

Netherlands

**Jos Van Pelt**

Belgium

**Joost Van Rosmalen**

Netherlands

**Michelle Van Ryn**

USA

**Peter Van Veldhuizen**

USA

**Marcel Van Vugt**

Netherlands

**Vincent Vander Poorten**

Belgium

**Greta Varchi**

Italy

**Marileila Varella-Garcia**

USA

**Andreas Varkaris**

USA

**John Varlotto**

USA

**Mohammad Vasei**

Iran

**Vasyl Vasko**

USA

**Theodoros Vassilakopoulos**

Greece

**Athanassios Vassilopoulos**

USA

**David Vaughn**

USA

**Peter Vaupel**

Germany

**Dominique Vaur**

France

**Mikhail Vaysberg**

USA

**Piret Veerus**

Estonia

**Vaneja Velenik**

Slovenia

**Sergia Velho**

Portugal

**Tiziana Venesio**

Italy

**Girish Venkataraman**

USA

**Marina Vercelli**

Italy

**Paolo Verderio**

Italy

**Douglas Vernimmen**

UK

**Maud Verstraete**

Belgium

**Andre Vettore**

Brazil

**Gustavo Viani**

Brazil

**Laura Vidal Boixader**

Spain

**Sergi Vidal Sicart**

Spain

**Andre F Vieira**

Portugal

**Giuseppe Viglietto**

Italy

**Riccardo Vigneri**

Italy

**Raffaella Villa**

Italy

**Priam Villalonga**

Spain

**Piroska Virag**

Romania

**Pauline Vissers**

Netherlands

**Bella Vivat**

UK

**Francisco Vizoso**

Spain

**Giuseppe Vizzielli**

Italy

**Panagiotis Vlachostergios**

USA

**Virginie Vlaeminck-Guillem**

France

**Arndt Vogel**

Germany

**Michael Von Bergwelt-Baildon**

Germany

**André O. Von Bueren**

Germany

**Jw Voncken**

Netherlands

**Adri Voogd**

Netherlands

**Dirk Vordermark**

Germany

**Hilke Vorwerk**

Germany

**Mark Voskoboynik**

UK

**Gerassimos Voutsinas**

Greece

**Semir Vranic**

Bosnia and Herzegovina

**David Lukas Wachter**

Germany

**Jamie Wagner**

USA

**Kay-Uwe Wagner**

USA

**Robert Wagner**

USA

**Quinten Waisfisz**

Netherlands

**Stefan Walenta**

Germany

**Jan Walewski**

Poland

**Jo Waller**

UK

**Markus Wallwiener**

Germany

**Guohui Wan**

USA

**Bin Wang**

USA

**Chunmei Wang**

USA

**Jaw-Yuan Wang**

Taiwan

**Endi Wang**

USA

**Fen Wang**

USA

**Jiucun Wang**

China

**Jianhua Wang**

China

**Zhen-Ning Wang**

China

**Qian Wang**

Australia

**Li Dong Wang**

China

**Lu-Hai Wang**

Taiwan

**Michael Wang**

Singapore

**Ruoning Wang**

USA

**Shengzhi Wang**

USA

**Shouju Wang**

China

**Kui Wang**

China

**Tony Wang**

USA

**Zhen Wang**

China

**Chen Wang**

USA

**Anhui Wang**

China

**Cheng-Ping Wang**

Taiwan

**Kai Wang**

China

**De-Shen Wang**

China

**Ji Ming Wang**

USA

**Guanghui Wang**

China

**Jiahong Wang**

China

**Wen-Lung Wang**

Taiwan

**Lei Wang**

USA

**Wei Wang**

USA

**Xiaoxia Wang**

China

**Xiao-Si Wang**

UK

**Zehua Wang**

China

**Irene Wapnir**

USA

**Alister Ward**

Australia

**Doug Ward**

UK

**John Ward**

USA

**Fredrik Warnberg**

Sweden

**Sugiko Watanabe**

Japan

**Naoki Watanabe**

Japan

**William Watson**

Ireland

**Elizabeth Wattenberg**

USA

**Patrik Weder**

Switzerland

**Zheng Wei**

China

**Joanne Weidhaas**

USA

**Kurt Weiss**

USA

**Aaron Weiss**

USA

**Meng Welliver**

USA

**Thilo Welsch**

Germany

**Shijun Wen**

China

**Thomas G. Wendt**

Germany

**Rhay-Hung Weng**

Taiwan

**Martin Werner**

Germany

**Haim Werner**

Israel

**Kristin Werner**

Germany

**Jelle Wesseling**

Netherlands

**Derek West**

USA

**Tamarah Westmoreland**

USA

**Brian Weston**

USA

**Nicholas Wetjen**

USA

**Chery Whipple**

USA

**Hayley Whitaker**

UK

**Emily White**

USA

**Alexandra White**

USA

**David Whiteman**

Australia

**Emilia Wiechec**

Sweden

**Armin Wiegering**

Germany

**Charlotte Wilhelm-Benartzi**

UK

**Stefan Willems**

Netherlands

**Basem William**

USA

**Anna-Lise Williamson**

South Africa

**Matthew Witek**

USA

**Sara Wobker**

USA

**Thomas Wolf**

Germany

**Anita Wolfer**

Switzerland

**Hendrik Andreas Wolff**

Germany

**Aaron H. Wolfson**

USA

**Younf-Joo Won**

South Korea

**Young-Woong Won**

South Korea

**Alice Wong**

Hong Kong

**Eric Wong**

USA

**Kwong-Kwok Wong**

USA

**Kwan Yeung Wong**

Hong Kong

**Maria Wong**

Hong Kong

**Shun Wong**

Canada

**Alex Wong**

USA

**Wendy Woodward**

USA

**Chadwick Wright**

USA

**Gerald Wright**

USA

**Qiang Wu**

China

**Anhua Wu**

China

**Cheng-Wen Wu**

Taiwan

**Erxi Wu**

USA

**Jun-Xin Wu**

China

**Ning Wu**

Canada

**Weizhong Wu**

China

**Jiong Wu**

China

**Junhua Wu**

China

**Minghua Wu**

China

**Xuping Wu**

Sweden

**Fen Xia**

USA

**Hong-Fei Xia**

China

**Jinglin Xia**

China

**Dong Xiao**

USA

**Feifei Xiao**

USA

**Guanghua Xiao**

USA

**Helan Xiao**

Canada

**Jianru Xiao**

China

**Xiangwei Xiao**

USA

**Feng Xiaobin**

China

**Xu Xinglu**

China

**Shunbin Xiong**

USA

**Fan Xu**

China

**Jia Xu**

USA

**Jiejie Xu**

China

**Yan Xu**

USA

**Yh Xu**

China

**Yaji Xu**

USA

**Yasuhide Yamada**

Japan

**Toshiyuki Yamaji**

Japan

**Yoriko Yamashita**

Japan

**Takashi Yamatodani**

Japan

**Takahiro Yamauchi**

Japan

**Hideya Yamazaki**

Japan

**Chunhong Yan**

USA

**Dongsheng Yan**

China

**Seung Ho Yang**

South Korea

**Haining Yang**

USA

**Jianhua Yang**

USA

**Jin-Ming Yang**

USA

**Lei Yang**

China

**Lily Yang**

USA

**Qiwei Yang**

USA

**Ping Yang**

China

**Jinji Yang**

China

**Ping-Chang Yang**

Canada

**Tsung-Lin Yang**

Taiwan

**Elizabeth Yanik**

USA

**Qizhi Yao**

USA

**Jian Yao**

Japan

**Slav Yartsev**

Canada

**Hemad Yasaei**

UK

**Patsy Yates**

Australia

**Ozan Yazici**

Turkey

**Yihong Ye**

USA

**Herman Yeger**

Canada

**Chau-Ting Yeh**

Taiwan

**Shiou-Hwei Yeh**

Taiwan

**Te-Huei Yeh**

Taiwan

**Chueh-Chuan Yen**

Taiwan

**Chia Jui Yen**

Taiwan

**Sriram Yennu**

USA

**Young Yeom**

South Korea

**Ho-Young Yhim**

South Korea

**He Yifeng**

China

**James Yiin**

USA

**John Yim**

USA

**Lu Yin**

USA

**Tinghui Yin**

China

**Jun Yin**

China

**Michele Yip-Schneider**

USA

**Takehiko Yokobori**

Japan

**Kohei Yokoi**

Japan

**Raymund Yong**

USA

**Bryan Yoo**

USA

**Kyong-Ah Yoon**

South Korea

**Go Yoshida**

Japan

**Junko Yoshida**

Japan

**Takaki Yoshikawa**

Japan

**Tiangeng You**

China

**Danny Youlden**

Australia

**Buhyun Youn**

South Korea

**Jessica Young**

USA

**Susan Yount**

USA

**Ramy Youssef**

USA

**Ramy Youssef Yaacoub**

USA

**Chao-Lan Yu**

USA

**Jun Yu Hong**

Kong

**Guanzhen Yu**

China

**Helena Yu**

USA

**Huiqing Yuan**

China

**Guangwen Yuan**

China

**Zheng Yuan**

China

**Yuan Yuan**

China

**Yuan Yuan**

USA

**Wei Yue**

USA

**Xiaoqiang Yue**

China

**Jong Won Yun**

South Korea

**Jason Yustein**

USA

**Aziz Zaanan**

France

**Iris Zachary**

USA

**Fabrizio Zanconati**

Italy

**Roberto Zanetti**

Italy

**Uwe Zangemeister-Wittke**

Switzerland

**Marjorie Zauderer**

USA

**Robert Zeillinger**

Austria

**Alain Zeimet**

Austria

**Zihua Zeng**

USA

**Jiping Zeng**

China

**Zhi Zeng**

China

**Zhirong Zeng**

China

**Pio Zeppa**

Italy

**Alessandro Zerbi**

Italy

**Baolin Zhang**

USA

**Faming Zhang**

China

**Hong (Amy) Zhang**

USA

**Jinsheng Zhang**

China

**Jun Zhang**

China

**Junran Zhang**

USA

**Ming Zhang**

USA

**Siyuan Zhang**

USA

**Weiguo Zhang**

USA

**Xiaoyu Zhang**

USA

**Kunhe Zhang**

China

**Yi Zhang**

USA

**Mei Zhang**

USA

**Guangjun Zhang**

China

**Guo Zhang**

China

**Lining Zhang**

China

**Lin Zhang**

USA

**Meixia Zhang**

China

**Mei-Yun Zhang**

Hong Kong

**Xuegong Zhang**

China

**Zhen Zhang**

USA

**Zhongzu Zhang**

China

**Zhi-Gang Zhang**

China

**Chen Zhao**

USA

**Qichen Zhao**

China

**Weili Zhao**

China

**Guoqiang Zhao**

China

**Hongyun Zhao**

China

**Minhua Zheng**

China

**Yongqiu Zheng**

USA

**Yuanyi Zheng**

China

**Qiaoming Zhi**

China

**Yuan Zhiyong**

China

**Weide Zhong**

China

**Bo Zhou**

China

**Qingyu Zhou**

USA

**Shu-Feng Zhou**

USA

**Tong Zhou**

USA

**Yu-Hao Zhou**

China

**Jingjiao Zhou**

USA

**Jinfeng Zhou**

China

**Jue-Yu Zhou**

China

**Yifeng Zhou**

China

**Jingde Zhu**

China

**Ji Zhu**

China

**Yueming Zhu**

USA

**Zhitu Zhu**

China

**Zhengping Zhuang**

USA

**Suzanna Zick**

USA

**Andries Zijlstra**

USA

**Volker Ziller**

Germany

**Michael Zilliox**

USA

**Yitzhak Zimmer**

Switzerland

**Daniel Zips**

Germany

**Mateo Ziu**

USA

**Oliver Zivanovic**

USA

**Inti Zlobec**

Switzerland

**Anna Zolkiewska**

USA

**Gabriele Zoppoli**

Italy

**Xiaoping Zou**

China

**Chiara Zuiani**

Italy

**Mingxin Zuo**

USA

**Wilbert Zwart**

Netherlands

**Ellen Zwarthoff**

Netherlands

**Krzysztof Zwierz**

Poland

**Norberto Walter Zwirner**

Argentina

